# Evolution of an Amniote-Specific Mechanism for Modulating Ubiquitin Signaling via Phosphoregulation of the E2 Enzyme UBE2D3

**DOI:** 10.1093/molbev/msaa060

**Published:** 2020-03-12

**Authors:** Monica Roman-Trufero, Constance M Ito, Conrado Pedebos, Indiana Magdalou, Yi-Fang Wang, Mohammad M Karimi, Benjamin Moyon, Zoe Webster, Aida di Gregorio, Veronique Azuara, Syma Khalid, Christian Speck, Tristan Rodriguez, Niall Dillon

**Affiliations:** m1 Gene Regulation and Chromatin Group, MRC London Institute of Medical Sciences, Imperial College London, Hammersmith Hospital Campus, London, United Kingdom; m2 Department of Chemistry, University of Southampton, Southampton, United Kingdom; m3 DNA Replication Group, Institute of Clinical Sciences, Imperial College London, Hammersmith Hospital Campus, London, United Kingdom; m4 Bioinformatics and Computing, MRC London Institute of Medical Sciences, Imperial College London, Hammersmith Hospital Campus, London, United Kingdom; m5 Transgenics and ES Cell Facility, MRC London Institute of Medical Sciences, Imperial College London, Hammersmith Hospital Campus, London, United Kingdom; m6 BHF Centre for Research Excellence, National Heart and Lung Institute, Imperial College London, Hammersmith Hospital Campus, London, United Kingdom; m7 Institute of Reproductive and Developmental Biology, Imperial College London, Hammersmith Hospital Campus, London, United Kingdom

**Keywords:** ubiquitination, amniote, evolution, embryogenesis, mouse

## Abstract

Genetic variation in the enzymes that catalyze posttranslational modification of proteins is a potentially important source of phenotypic variation during evolution. Ubiquitination is one such modification that affects turnover of virtually all of the proteins in the cell in addition to roles in signaling and epigenetic regulation. UBE2D3 is a promiscuous E2 enzyme, which acts as an ubiquitin donor for E3 ligases that catalyze ubiquitination of developmentally important proteins. We have used protein sequence comparison of UBE2D3 orthologs to identify a position in the C-terminal α-helical region of UBE2D3 that is occupied by a conserved serine in amniotes and by alanine in anamniote vertebrate and invertebrate lineages. Acquisition of the serine (S138) in the common ancestor to modern amniotes created a phosphorylation site for Aurora B. Phosphorylation of S138 disrupts the structure of UBE2D3 and reduces the level of the protein in mouse embryonic stem cells (ESCs). Substitution of S138 with the anamniote alanine (S138A) increases the level of UBE2D3 in ESCs as well as being a gain of function early embryonic lethal mutation in mice. When mutant S138A ESCs were differentiated into extraembryonic primitive endoderm, levels of the PDGFRα and FGFR1 receptor tyrosine kinases were reduced and primitive endoderm differentiation was compromised. Proximity ligation analysis showed increased interaction between UBE2D3 and the E3 ligase CBL and between CBL and the receptor tyrosine kinases. Our results identify a sequence change that altered the ubiquitination landscape at the base of the amniote lineage with potential effects on amniote biology and evolution.

## Introduction

Ubiquitination is an ancient posttranslational modification that originated in Archaea (Grau-Bove et al. 2015) and controls a wide range of cellular and developmental processes across eukaryotic lineages (reviewed by Rape [2018]). One of the major functions of ubiquitination is protein quality control and regulation of the rate of turnover of individual proteins. This occurs through the addition of polyubiquitin chains, which act as signals that direct proteins to the proteasome for degradation. In addition to these functions, ubiquitination is involved in diverse processes that include epigenetic regulation of transcription, control of lysosomal trafficking of growth factor receptors, regulation of DNA repair, and modulation of signaling pathways including, among others, the MEK/ERK, NF-κB, and Wnt signaling pathways. These different types of regulation can occur through a variety of ubiquitination events including monoubiquitination of specific residues and addition of polyubiquitin chains with different topologies that are generated by varying combinations of ubiquitin chain linkages (Rape 2018).

Ubiquitin is first activated by formation of a thioester bond with the E1 enzyme and is then transferred to an E2 ubiquitin-conjugating enzyme. The E2 enzyme acts as the donor for transfer to the substrate, a reaction that is catalyzed by the action of an E3 ubiquitin ligase, which is the component of the cascade that confers most of the substrate specificity. Mammals have 1–2 E1 enzymes, around 30 E2 enzymes and at least 600 E3 ligases. This means that each E2 generally interacts with multiple E3 enzymes. Together with the fact that different E2s preferentially give rise to ubiquitin chains with different linkages (Ye and Rape 2009), this gives E2 enzymes a broad potential for influencing cellular functions that has still to be explored in depth.

UBE2D3 is a promiscuous E2 enzyme that acts as the donor for a number of E3 ligases that are involved in protein quality control and important signaling and regulatory pathways (Jensen et al. 1995; Brzovic and Klevit 2006; Wenzel et al. 2011). The UBE2D family members are the vertebrate orthologs of the *Drosophila melanogaster* Effete (Eff) protein (also known as UBCD1), which shares 94% identity with human UBE2D3 and has been shown to have multiple roles in *Drosophila* development (Chen et al. 2009; Cipressa and Cenci 2013).

Among the E3 ligases that use UBE2D3 as an ubiquitin donor in vertebrates is the protein encoded by the developmentally important *CBL* protooncogene (Liyasova et al. 2019), which is responsible for controlling the endocytosis and lysosomal trafficking of the epidermal growth factor receptor (Fortian et al. 2015) and ubiquitination and degradation of platelet-derived growth factor receptor-α (PDGFRα) and the fibroblast growth factor receptors (FGFRs) (Vantler et al. 2006; Haugsten et al. 2008). Other E3 ligases for which UBE2D enzymes have been shown to act as ubiquitin donors in vertebrates include the Polycomb protein Ring1B (Bentley et al. 2011), which catalyzes monoubiquitination of histone H2AK119, MDM2, which ubiquitinates the tumor suppressor and checkpoint protein p53 and regulates its turnover (Saville et al. 2004), and CHIP/STUB1, which plays an essential role in protein turnover and quality control (VanPelt and Page 2017).

Acquisition of the capacity to live entirely on land was a key event in vertebrate evolution that took place during the Carboniferous period. The transition occurred when tetrapod amphibia that had evolved in the Devonian acquired the ability to breed on dry land without returning to water, leading to evolution of the amniote lineage (reviewed by Clack [2012]). One of the major changes that led to this capability was the development of extraembryonic membranes, which surrounded the amniote embryo, protecting it from dessication (Ferner and Mess 2011; Stern and Downs 2012). The first true amniote fossil has been dated to 314 Ma (Carroll 1964), and the common ancestor to amniotes is thought to have lived between 340 and 314 Ma (Clack 2012). Other changes that made a terrestrial existence possible included skeletal alterations that allowed movement and feeding on land (Clack 2012) and the physiological changes that made it possible for amniotes to become fully air breathing. The detailed molecular mechanisms that led to these changes are still poorly understood but they are likely to have included a mixture of changes to transcription factor–mediated control of developmental genes and to the signaling pathways that control development and organogenesis.

Here, we describe an unusual mutation that occurred in the common ancestor to modern amniotes generating a single amino acid change at a highly conserved site in the UBE2D3 protein. The substituted serine (S138) is completely invariant across amniote lineages, whereas the position is occupied by a conserved alanine in anamniote vertebrates, invertebrates, and single-celled eukaryotes. We show that phosphorylation of S138 by Aurora B kinase disrupts the structure of UBE2D3, destabilizing it, and reducing its level and activity. The reduction in UBE2D3 activity affects the functioning of the CBL E3 ligase, increasing the expression of receptor tyrosine kinases (RTKs) in differentiating extraembryonic primitive endoderm (PrE). Mutation of the S138 residue to the anamniote alanine has a gain of function effect that results in early embryonic lethality in mouse embryos, compromised ability of mutant ES cells (ESCs) to develop into PrE and reduced levels of PDGFRα and FGFR1 in differentiating PrE. Our results identify a novel regulatory pathway that originated in the common ancestor to modern amniotes and affected the ubiquitination landscape with potential impacts on amniote evolution.

## Results

### Comparison of UBE2D3 Orthologs across Eukaryotic Lineages

**Fig. 1.**
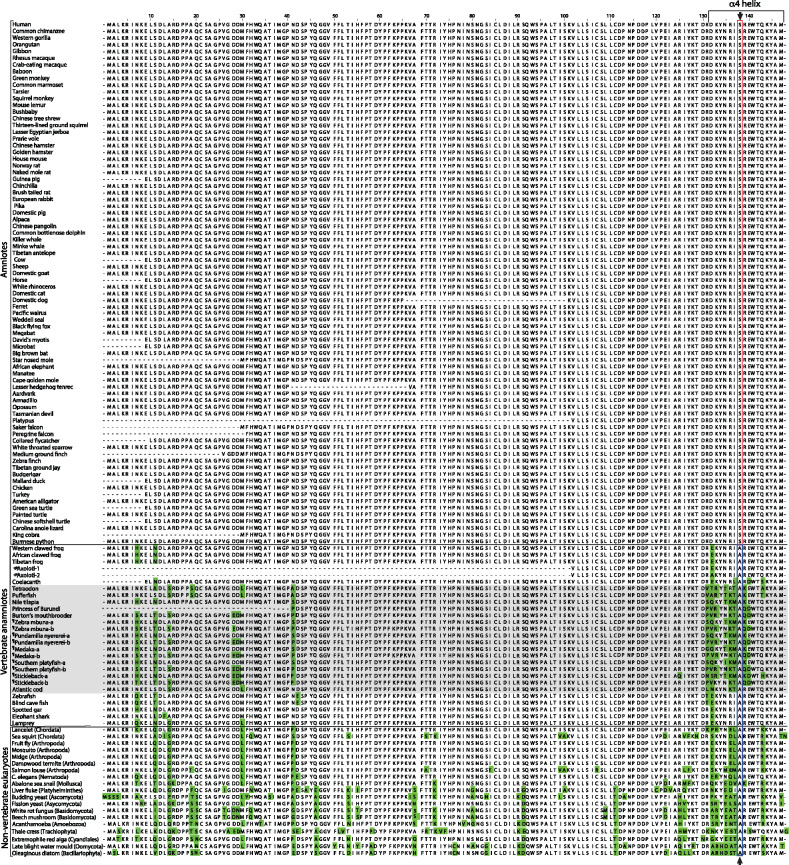
Alignment of the sequences of vertebrate *Ube2d3* genes and nonvertebrate *Ube2d3* orthologs. Numbering starts from the first residue of the human sequence. Residues that differ from the human sequence are highlighted in green with the exception of position 138. The serine and alanine residues at position 138 are indicated by red and yellow boxes, respectively, and by vertical arrows above and below the sequence. Dashed lines indicate gaps in some of the available sequences. Full species names and accession numbers and sources for the sequences are provided in supplementary tables 1 and 2, Supplementary Material online. #The Axolotl Exon-6 and exon-7 sequences were located for two UBE2D genes by blast searching the Axolotl genome sequence (for details, see supplementary Methods, Supplementary Material online). Sequence comparison indicated that these genes were UBE2D2 or UBE2D3, but it was not possible to determine which is UBE2D3. Therefore, both sequences are shown as Axolotl-1 and -2. The sequences differ by a single residue (position 132), which is occupied by E or A. ^¶^Duplicated *Ube2d3* genes (*Ube2d3*-a and *Ube2d3*-b) arising from the whole-genome duplication that occurred in the common ancestor to teleost fish (see supplementary Discussion, Supplementary Material online).

We chose to carry out a comparative sequence analysis of UBE2D3 orthologs because of previous evidence that the activity of UBE2D3 is regulated by phosphorylation (Frangini et al. 2013) and because it is the most highly expressed member of the conserved UBE2D family of ubiquitin-conjugating enzymes (Jensen et al. 1995; Liyasova et al. 2019), which have the capacity to interact with a large number of E3 enzymes (Brzovic and Klevit 2006). The sequences of UBE2D3 orthologs from 118 eukaryotic species were compared using the human sequence as a reference (fig. 1 and supplementary table 1, Supplementary Material online) (see Materials and Methods for details). The results show a very high level of conservation, with 94% sequence identity between humans and *Drosophila melanogaster* and >80% identity observed between humans and basal eukaryotes such as the oleaginous diatom, *Fistuilifera solaris*, and the extremophilic red alga, *Galdieria sulphuria* (fig. 1 and supplementary table 1, Supplementary Material online). As expected, the overall variation relative to human UBE2D3 increases with increasing evolutionary distance but the variation is nonrandom and confined to specific residues or short regions, whereas significant stretches of the protein are completely invariant. Many of the substitutions observed are conservative and, even where the substitution is nonconservative the variation is generally restricted to a few amino acids. This suggests that the variable regions have also been subject to significant functional constraint during evolution.

The highest variability in the UBE2D3 sequence is observed in a stretch of 20 residues (127–147) that contains an α-helix at the C-terminal end of the protein (fig. 1 and supplementary fig. S1 and table 1, Supplementary Material online). The variation within the C-terminal region is also nonrandom. For example, basal fish lineages (lamprey, elephant shark, and spotted gar) show 90% homology between this region and the equivalent region of human UBE2D3, whereas in a subgroup of teleost fish, the Acanthomorpha, the homology ranges from 55% to 75% and is lower than the homology between humans and the majority of invertebrate metazoan species examined (fig. 1 and supplementary table 1, Supplementary Material online, gray shading). The Acanthomorpha (spiny rayed fishes) are a successful and morphologically diverse fish taxon that first appeared ∼120 Ma and comprise around one-third of all living vertebrates (Friedman 2010; Chen et al. 2014; Bellwood et al. 2015). They include the pufferfish, cichlids, sticklebacks, and seahorse as well as cod and sea bass. Several of the Acanthomorph species examined had gene duplications that are likely to be a product of the whole-genome duplication that occurred in the common ancestor to teleosts (Meyer and Van de Peer 2005; Opazo et al. 2013). Neofunctionalization or subfunctionalization of these duplicated genes could have contributed to the increased variation in the C-terminal region of Acanthomorphs. It is notable that the side chains of the hypervariable amino acids are mainly located on the outward facing surface of the α4-helix, whereas the invariant residues mostly face inward toward the hydrophobic core of the protein (supplementary fig. S1*B*, Supplementary Material online). This suggests that the variable residues are involved in protein–protein contacts and that these contacts have been subject to evolutionary change in the Acanthomorpha. Additional discussion of the results obtained from the analysis of fish sequences can be found in supplementary materials, Supplementary Material online.

### Evidence of an A138S Substitution in the Common Ancestor to Amniotes

A further striking feature of the C-terminal region of UBE2D3 that was revealed by the sequence comparison was the very high conservation of the alanine residue at position 138 (A138) in anamniotes, invertebrates, and single-celled eukaryotes (fig. 1 and supplementary table 1, Supplementary Material online). The residue occupying position 138 is located in the center of the C-terminal α4-helix (supplementary fig. S1, Supplementary Material online) and the conservation of alanine at this position is maintained even in the Acanthamorph fish, where the residues on both sides of position 138 are highly variable. However, in all amniote species examined, this position is occupied by serine (S138), a residue that is not found at position 138 in any of the other eukaryotic species examined. This leads to the conclusion that an alanine to serine substitution occurred in the common ancestor to modern amniotes and that the S138 residue has been conserved in all amniote lineages. The uniqueness of the alanine to serine substitution in UBE2D3 is further supported by a comparative sequence analysis of the closely related UBE2D2 and UBE2D1 enzymes that showed that both of these enzymes have alanine as a conserved residue at position 138 in amniotes (supplementary tables 3 and 4, Supplementary Material online). The sequence context around UBE2D3-S138 means that the substitution created a consensus phosphorylation site for the Aurora B kinase that is specific to amniotes, and we have previously reported that UBE2D3-S138 is phosphorylated in vitro and in vivo by Aurora B (Frangini et al. 2013). The acquisition of the serine at a critical point in vertebrate evolution and its absolute conservation in amniotes raised the possibility that the A138S substitution played a functional role in amniote evolution.

### Mutation of S138 to the Anamniote Alanine Increases the Stability of UBE2D3 in ESCs

Analysis of the mRNA levels of UBE2D family members in pluripotent mouse ESCs showed that UBE2D3 is the predominantly expressed UBE2D enzyme in these cells with levels for UBE2D2 and UBE2D1 mRNA more than 15-fold lower than for UBE2D3 (supplementary fig. S2*A*, Supplementary Material online). This allowed us to investigate the functional roles of the UBE2D3-S138 residue in the embryonic cell lineages of a model amniote by replacing it with the anamniote alanine residue in mouse ESCs using CRISPR/Cas9. Following transfection of ESCs with the guide RNA (gRNA), Cas9 expression construct and targeting sequence (see Methods), clones were isolated and genotyped by nested polymerase chain reaction (PCR). Out of a total of 37 clones analyzed, 10 were homozygous for the mutation. The mutant cells grew normally and showed no overt changes in cell cycle profile (supplementary fig. S2*B*, Supplementary Material online). To ensure that any differences in the behavior of the cells were caused by the mutation, CRISPR/Cas9 mutagenesis was used to generate a revertant cell line from one mutant clone. For subsequent functional analysis of the ESCs, wild-type ESCs were compared with the S138A mutant clone and the A138S revertant that was generated from it.

When the mutant UBE2D3 protein was analyzed by western blotting of protein extracts from the UBE2D3-S138A ESCs, it became apparent that the presence of the S138A mutation enhanced the stability of the protein (supplementary fig. S2*A*, Supplementary Material online). The mean level of UBE2D3 was increased by ∼3-fold (*P* < 0.05, *n* = 5). Analysis of *Ube2D3* mRNA levels showed no significant change in the transcript levels in the mutant and revertant cells compared with wild-type cells (supplementary fig. S2*C*, Supplementary Material online). Inhibition of protein synthesis by incubation of wild-type and mutant cells with cycloheximide showed a significantly lower rate of degradation of UBE2D3-S138A compared with wild-type UBE2D3 after 5 h of cycloheximide treatment, confirming the increased stability of the mutant protein (supplementary fig. S2*B*, Supplementary Material online). Our findings provide evidence that the alanine to serine substitution at position 138 substantially affected the stability of UBE2D3, thereby altering the functioning of the protein in amniotes.

### Phosphorylation of UBE2D3-S138 by Aurora B Destabilizes the Protein in ESCs

**Fig. 2.**
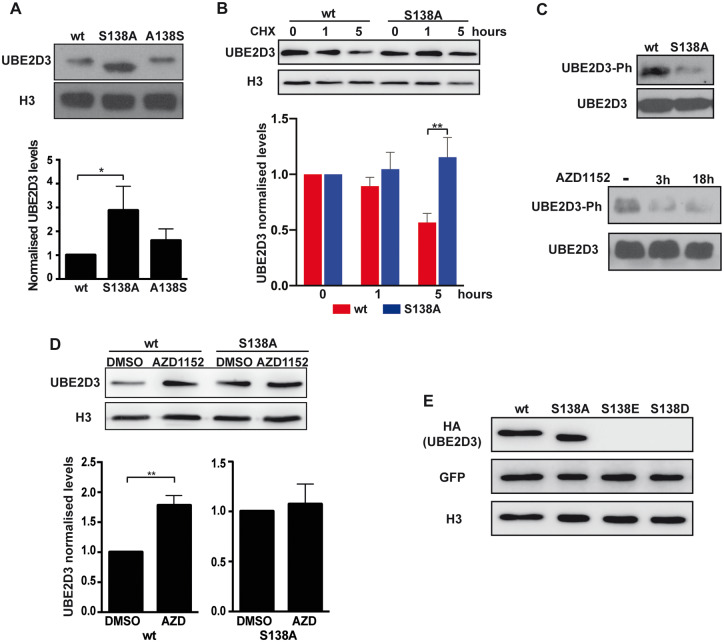
Characterization of *Ube2d3*-S138A mutant ESCs. (*A*) Western blot analysis of UBE2D3 levels in wt, S138A mutant, and A138S revertant ESCs. Top panel: representative blot. Bottom panel: quantification of band intensity relative to histone H3; mean ± SEM, *n* = 5; *P* values calculated by *t-*test for this, (*B*) and (*D*): *P < 0.05 and **P < 0.01. (*B*) Effect of protein synthesis inhibition on UBE2D3 levels: western blot (top panel) and quantification (bottom panel) of UBE2D3 levels in wt and S138A mutant cells after treatment for 1 and 5 h with 50 μg/ml cycloheximide (CHX). (*C*) Phosphorylation of UBE2D3-S138 in ESCs. Top panel: western blot of extracts from wt and *Ube2d3*-S138A ESCs with anti-phospho-S138-UBE2D3 (UBE2D3-Ph) and anti-UBE2D3 (UBE2D3) antibodies. To allow direct comparison of the UBE2D3-Ph bands, samples were adjusted so that equivalent levels of total UBE2D3 were loaded. Bottom panel: western blot of extracts from wt ESCs treated with Aurora B inhibitor AZD1152 for 3 and 18 h and probed with anti-phospho-UBE2D3. (*D*) Western blot analysis of UBE2D3 levels in wt and S138A mutant ESCs treated for 3 h with Aurora B inhibitor AZD1152 or vehicle (DMSO). Propidium iodide staining and FACS analysis showed that AZD1152 had no effect on the cell cycle (data not shown). Top panel: representative blot. Bottom panel quantification relative to histone H3, mean ± SEM, *n* = 4. (*E*) Western blot analysis of wt, phosphomutant S138A, and phosphomimetics S138E and S138D mutants expressed in ESC. Cells were sorted for expression of the IRES-GFP after the transient transfection.

Phosphorylation of UBE2D3-S138 (Frangini et al. 2013) has the potential to affect the stability of the protein by introducing a negative charge in the center of the α4-helix of the protein. The presence of phosphorylated UBE2D3 in ESCs was demonstrated using an antibody that specifically recognizes the phosphorylated UBE2D3-S138 residue (Frangini et al. 2013) (anti-UBE2D3-Ph; supplementary fig. 2*D*, Supplementary Material online). Western blotting of protein extracts from wild-type and UBE2D3-S138A mutant ESCs using the anti-UBE2D3-Ph antibody gave a strong band in wild-type ESCs and only a low background band in the S138A mutant cells (fig. 2*C*, top panel). The background is likely to result from cross-reaction of the anti-UBE2D3-Ph antibody with the unphosphorylated protein that we have observed in vitro. Incubation of ESCs for 3 and 18 h with the specific Aurora B inhibitor AZD1152 reduced the level of phosphorylated UBE2D3 to background levels (fig. 2*C*, bottom panel), confirming the involvement of Aurora B in phosphorylating S138. These results show that Aurora B has a major role in phosphorylating wild-type UBE2D3-S138 in ESCs, although we cannot completely exclude the possibility that S138 is also a target for phosphorylation by other kinases.

To directly test whether phosphorylation of UBE2D3-S138 destabilizes UBE2D3, wild-type and UBE2D3-S138A mutant ESCs were incubated for 3 h with the specific Aurora B inhibitor AZD1152. The short incubation period was used to avoid any possibility of interference with the cell cycle. Western blot analysis showed that inhibition of Aurora B led to a significant increase in the level of UBE2D3 protein compared with cells treated with vehicle (fig. 2*D*, left panel). In contrast, no change was observed in the level of the UBE2D3-S138A mutant protein, where the Aurora B phosphorylation site is not present. Treatment of wild-type ESCs with the proteasome inhibitor MG132 resulted in a strong increase in the level of UBE2D3 (supplementary fig. S5*A*, Supplementary Material online), indicating that the protein is being degraded by the proteasome. As a further test of the effect of phosphorylation of S138, phosphomimetic mutant UBE2D3 proteins that had glutamic acid or aspartic acid residues at position 138 were transiently expressed in wild-type ESCs. The results show that the phosphomimetic mutants are very unstable in ESCs (fig. 2*E*), providing further confirmation of the destabilizing effect of phosphorylation of S138.

### UBE2D3-S138 Phosphorylation Alters the Structure of the Protein Causing It to Become Insoluble and Aggregate

**Fig. 3.**
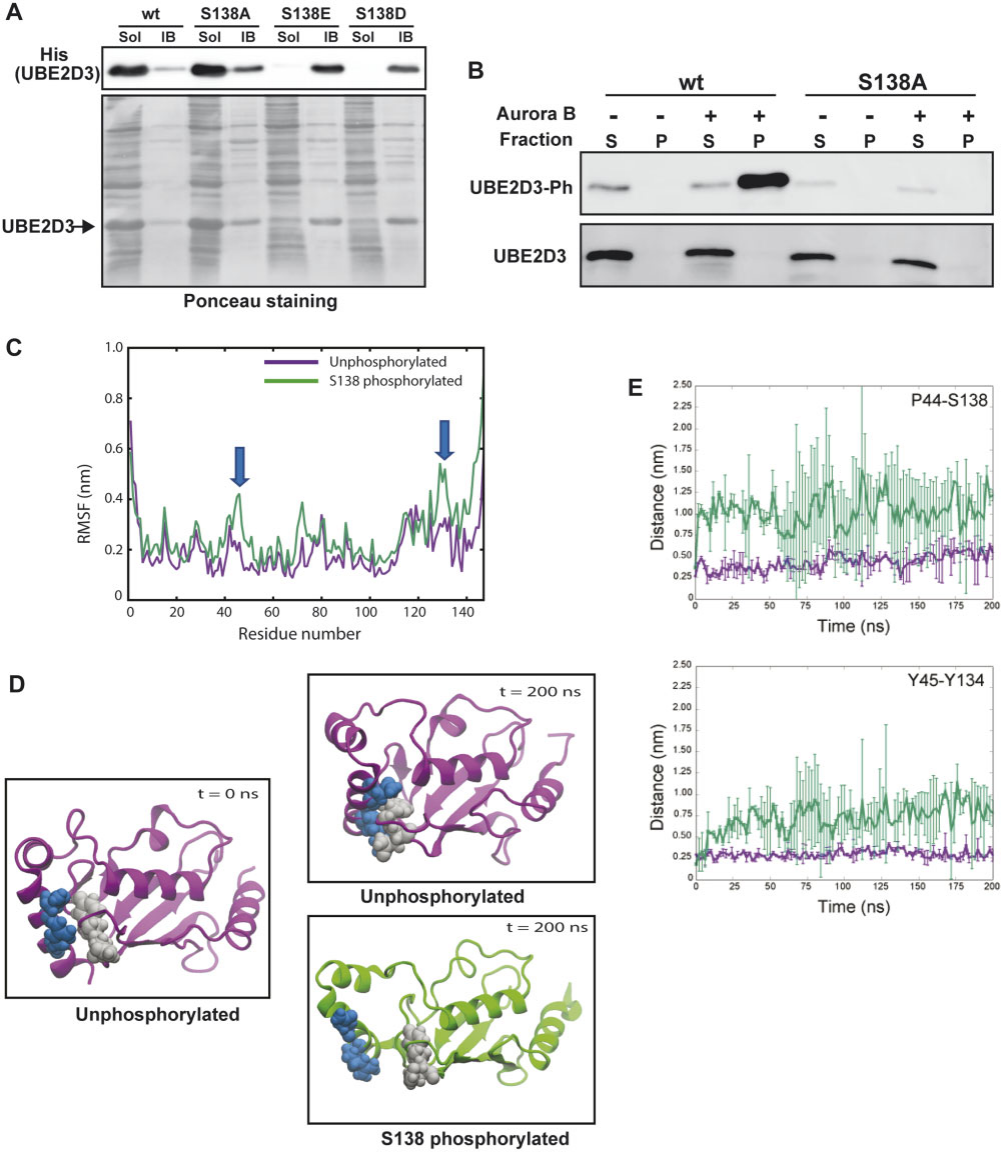
Effect of S138 phosphorylation on the structure of UBE2D3. (*A*) Solubility of UBE2D3 wt, phosphomutant S138A, and phosphomimetic S138E and S138D variants. UBE2D3 was expressed in bacteria and extracted as described in Materials and Methods. Soluble (Sol) and insoluble inclusion body (IB) fractions were analyzed by western blot and probed with anti-His antibody. Ponceau Red staining was used as loading control. (*B*) Phosphorylation of UBE2D3 at S138 causes precipitation. Western blot showing bacterially expressed and purified wt and S138A proteins after in vitro phosphorylation and centrifugation, S = supernatant, P = pellet. (*C*–*E*) Results of molecular dynamics simulation of the effects of S138 phosphorylation on the structure of UBE2D3. (*C*) Root mean square fluctuation calculations for unphosphorylated and S138 phosphorylated UBE2D3. Blue arrows indicate regions of increased mobility in the C-terminal α4-helix where S138 is located and around P44. (*D*) Effect of S138 phosphorylation on the positioning of the C-terminal α4-helix. P44 and Y45 are shown in gray and Y134 and S138 in blue. The unphosphorylated protein is purple, and the S138 phosphorylated protein is green. In the S138 phosphorylated protein, the α4-helix has moved away from the loop carrying P44 and Y45, exposing the hydrophobic core of the protein. (*E*) Distance between key residues in the simulations of the wild-type (purple) and phosphorylated (green) proteins. The distances were measured between P44 and S138 (top panel) and between Y45 and Y134 (bottom panel). Each curve is averaged over two independent simulations. The bars indicate the standard deviations.

To further investigate the effect of phosphorylation on UBE2D3, the wild-type, S138A, and phosphomimetic S138E and S138D proteins were expressed in bacteria with the aim of purifying the proteins for structural analysis (see Materials and Methods). A western blot of the soluble fractions and the pelleted inclusion body fractions obtained after Isopropyl β- d-1-thiogalactopyranoside (IPTG) induction of expression followed by lysis of the bacteria is shown in figure 3*A*. The bulk of the wild-type and S138A mutant UBE2D3 proteins was located in the soluble fractions, whereas the phosphomimetic mutant proteins were almost entirely localized to the insoluble inclusion bodies, providing direct evidence that the presence of a negatively charged residue at position 138 disrupts the structure of UBE2D3. This observation also indicates that the C-terminal α-helix has an important role in regulating the stability and level of the protein.

Efforts to purify soluble UBE2D3-S138E or S138D proteins by varying the bacterial growth conditions were unsuccessful, precluding direct functional analysis of the phosphomimetic mutant proteins. Therefore, as an alternative strategy, purified wild-type and UBE2D3-S138A proteins were incubated with Aurora B kinase and ATP. This was followed by centrifugation of the reaction mix and analysis of the supernatant and pelleted fractions by western blotting and probing with the anti-UBE2D3-S138Ph antibody. The results show clearly that the phosphorylated form of wild-type UBE2D3 is located almost entirely in the pelleted fraction (fig. 3*B*). No phosphorylation signal above background was observed in the pelleted or supernatant fractions for the UBE2D3-S138A mutant protein. Together with the observation that the phosphomimetic mutant proteins are insoluble when expressed in bacteria, these results provide strong evidence that phosphorylation of UBE2D3-S138 disrupts the structure of the protein, causing it to aggregate and become insoluble.

The effect of S138 phosphorylation on the molecular structure of UBE2D3 was also addressed by carrying out molecular dynamic simulations on the protein in the unphosphorylated and phosphorylated states (fig. 3*C*–*E*; see Materials and Methods). The results show that phosphorylation substantially alters the structure of the protein. Two independent simulations each of duration 200 ns were performed for the wild-type and phosphorylated protein. Although both proteins remained stable in terms of secondary structure on the timescale of the simulations, distinct differences in their conformational behavior were observed. The unphosphorylated protein displayed much less flexibility compared with the phosphorylated protein. This is clearly evident from the root mean square fluctuation, which measures the deviations from the average structure calculated over the simulation (fig. 3*C*). In particular, residues 43–55, 70–80, and 130–138 show a marked increase in flexibility in the phosphorylated compared with the unphosphorylated protein.

Visual inspection of the trajectories (fig. 3*D*) revealed that upon phosphorylation, a distinct conformational rearrangement is observed in the phosphorylated protein, namely that the α4-helix, which contains the phosphorylated S138 moves away from the loop carrying P44 and Y45, thereby exposing the hydrophobic core of the protein. This was further confirmed by calculating the distances between residues P44 and S138 and between Y45 and Y134, which were found to increase in the phosphorylated protein (fig. 3*E*). These results suggest that upon extension of the simulations, the phosphorylated protein is likely to begin to unfold. This rearrangement was not observed in the unphosphorylated protein, in which the α4-helix and the aforementioned loop remain in proximity throughout the simulations.

### Early Embryonic Lethal Phenotype of the UBE2D3-S138A Mutation in Mice

**Fig. 4.**
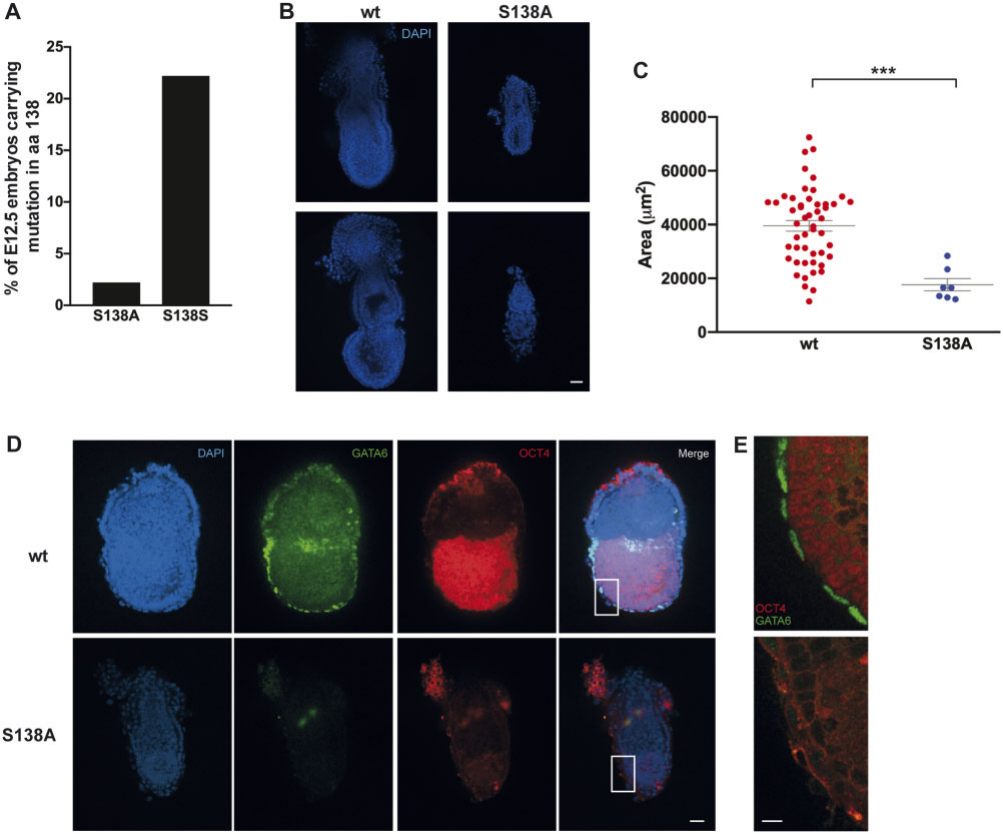
Early embryonic lethality of the *Ube2d3*-S138A mutation in mice. (*A*) Comparison of *Ube2d3*-S138A and -S138S mutation efficiency in E12.5 embryos obtained by CRISPR/Cas9 injection of fertilized eggs. The *x*-axis shows the percentage of embryos carrying the S138A or S138S mutation in amino acid (aa) 138 of *Ube2d3*. (*B*) Representative wt and mutant S138A E6.5 embryos. Scale bar = 50 μm. (*C*) Comparison of sizes for wt (*n* = 50) and S138A (*n* = 7) E6.5 embryos. The middle plane of each embryo was chosen for measurement; *P* value was calculated by Mann–Whitney *U* test (***P < 0.0001). (*D*) Wt and S138A E6.5 embryos stained with anti-OCT4 (epiblast) and anti-GATA6 (VE). Scale bar = 30 μm. (*E*) Higher magnification of boxed region in (*D*). Scale bar = 5 μm.

The evidence that phosphorylation downregulates the level of UBE2D3 in ESCs by disrupting the structure of the protein led us to consider the possibility that S138 phosphorylation could have acquired functional roles in embryogenesis during amniote evolution. To investigate the possible involvement of UBE2D3-S138 phosphorylation in amniote embryogenesis, we used the mouse embryo as a model system. The CRISPR/Cas9 targeting strategy described above was used to mutate S138 to the anamniote alanine in fertilized mouse eggs (see Materials and Methods). Initially, the eggs were implanted into foster mothers and allowed to develop to term. However, this did not yield any live mice that carried the mutation, which suggested that the S138A mutation is embryonic lethal in mice. To determine whether this is the case, embryos generated by injection of fertilized eggs were dissected at 12.5 days after injection and reimplantation of the eggs (E12.5) and five tissues were dissected from each embryo and analyzed by PCR to genotype the embryos and assess mosaicism (see Materials and Methods). The percentage of embryos carrying the S138A mutation at E12.5 was compared with the rate obtained by injection of a synonymous mutation (S138S) targeting sequence that alters the serine codon sequence, leaving the protein sequence unchanged (see Materials and Methods). Injection of the control S138S targeting sequence gave a mutation rate of 22% (10/45). In contrast, when the S138A targeting sequence was injected, 2% of E12.5 embryos (3/137) were positive for the mutation (fig. 4*A*). One of the three positive embryos obtained for the S138A mutation was dead, and sequencing of the two live embryos showed evidence of additional mutations around the splice site upstream from the mutated codon 138 that could have compensated for the S138A mutation by downregulating expression of the *Ube2D3* gene (data not shown). As CRISPR/Cas9 targeting is known to occasionally introduce unwanted mutations close to the targeting site, the observation that a small proportion of embryos were able escape from the effects of a gain of function mutation due to acquisition of additional compensating mutations was not unexpected. The 10-fold reduction in the proportion of E12.5 embryos carrying the S138A mutation compared with the targeting efficiency for S138S provides clear evidence that the S138A mutation has a high penetrance embryonic lethal effect in mice that affects the viability of the early embryo.

To further investigate the timing of the lethal effect, embryos were dissected 6.5 days after injection of the S138A targeting construct (E6.5) and analyzed by microscopy. This was followed by PCR analysis to determine genotype (see Materials and Methods for details). Seven homozygous embryos were identified at E6.5 and confirmed by sequencing (supplementary fig. S3, Supplementary Material online). A significant reduction in size was observed in the homozygous mutant S138A embryos compared with the wild-type embryos (*P* < 0.0001) (fig. 4*B* and *C*). Embryos were also stained with anti-GATA6 and anti-OCT4 antibodies to assess visceral endoderm (VE) and epiblast development. Staining of the homozygous mutant E6.5 embryos gave no staining for GATA6 (VE) and very weak staining for OCT4 (epiblast) compared with wild-type cells (fig. 4*D* and *E*) The close-up view of the peripheral region of the embryo shown in figure 4*E* reveals the absence of the GATA6 staining that is indicative of VE cells at this stage of development. These results suggest that the presence of alanine at position 138 of UBE2D3 interferes with epiblast expansion and differentiation of VE in mouse embryos between E4.5 and E6.5.

In summary, the CRISPR/Cas9 mutagenesis in mice shows that mutation of UBE2D3-S138 to the alanine that is conserved in nonamniote eukaryotes is a high penetrance early embryonic lethal mutation in mice with death most likely caused by a failure of lineage commitment and cellular expansion between E4.5 and E7.5. These results support the idea that phosphorylation of UBE2D3-S138 acquired important functional roles in embryogenesis during amniote evolution.

### Phosphorylation of UBE2D3-S138A Affects Differentiation of Embryonic PrE Cells

To investigate the role of UBE2D3-S138 phosphorylation in the early stages of lineage commitment in the mouse embryo, we used ESC differentiation to analyze the effect of the S138A mutation on the formation of the major embryonic cell lineages. An early event in embryonic development is the differentiation of a subset of inner cell mass cells in the blastocyst to give rise to PrE. The cells of the PrE lineage give rise to the parietal endoderm on the inner surface of the yolk sac, an extraembryonic membrane that plays a key role in nutrient exchange in amniote embryos (Ferner and Mess 2011; Sheng and Foley 2012). PrE cells are also precursors of VE, which has important inductive roles during amniote gastrulation (Stern and Downs 2012). VE was found to be largely absent from homozygous *Ube2d3*-S138A mouse embryos at E6.5 (fig. 4*E*).

**Fig. 5.**
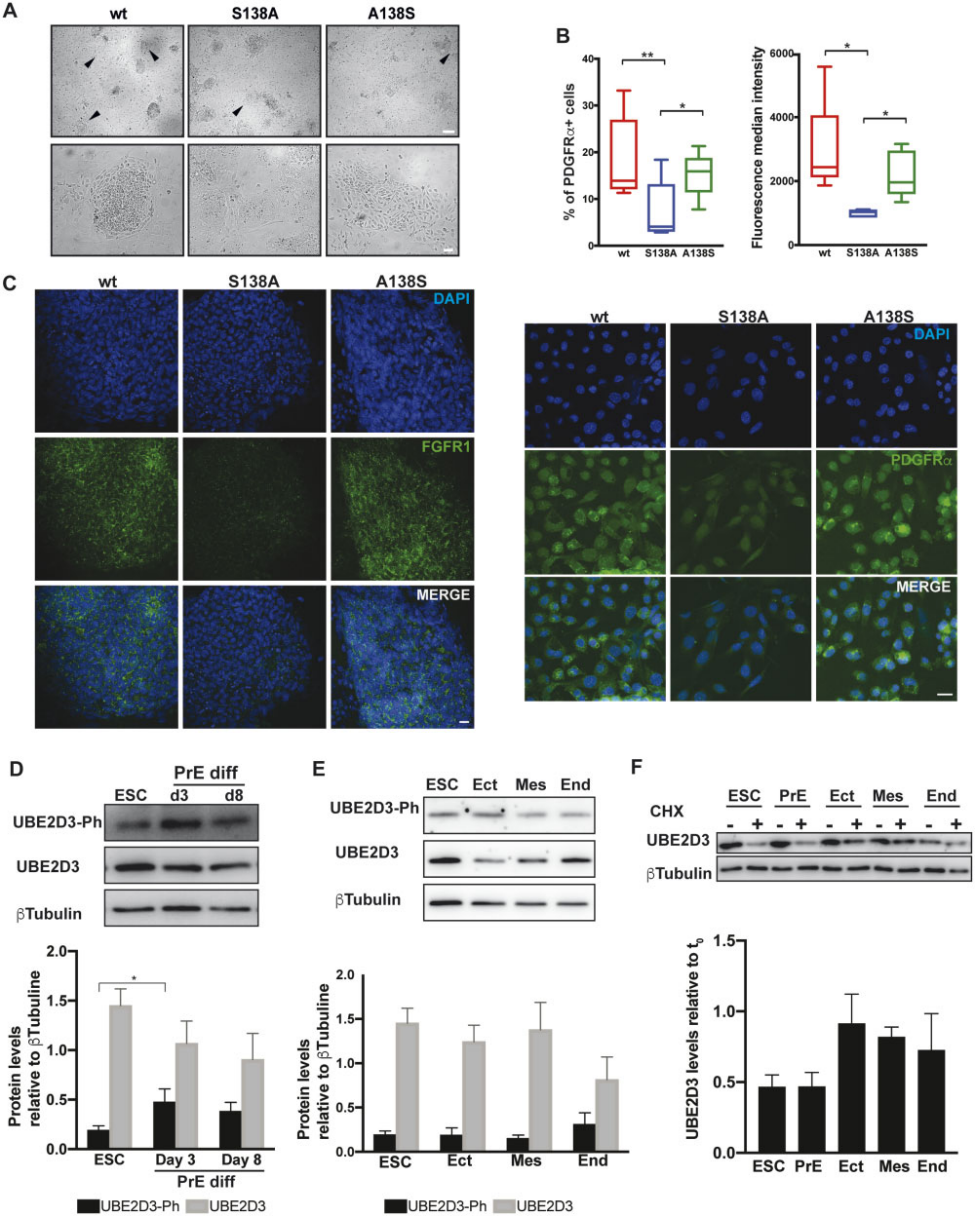
Impaired differentiation of *Ube2d3*-S138A mutant ESCs into XEN cells. (*A*) ESC differentiation into XEN cells. Bright-field images of wt, S138A mutant, and A138S revertant cells at day 8 of differentiation; arrows indicate colonies with XEN cell morphology. Top panel scale bar = 500 μm, bottom panel scale bar = 100 μm. (*B*) Cells collected at day 8 of XEN cell differentiation were stained with anti-PDGFRα and analyzed by FACS and immunostaining. Top left panel: percentage of cells that stained positive for PDGFRα. Top right panel: measurement of fluorescence median intensity of staining of cells that are positive for PDGFRα (*P* values calculated by *t*-test: *P < 0.05 and **P < 0.01, *n* = 5). Bottom panel: immunostaining of wt, S183A, and revertant cells with anti-PDGFRα. (*C*) Immunostaining of wt, S183A, and revertant cells with anti-FGFR1 on day 8 of differentiation to XEN cells. (*D* and *E*) Western blot analysis of UBE2D3-S138Ph and UBE2D3 levels in undifferentiated and differentiated ESCs. Levels of UBE2D3-S138ph and UBE2D3 were analyzed after 3 and 8 days of PrE differentiation (*D*) and in differentiated ectoderm, mesoderm, and endoderm (*E*). Bottom panels show quantification of UBE2D3-S138Ph and UBE2D3 levels relative to β-tubulin (mean ± SEM, *n* = 3). (*F*) Effect of protein synthesis inhibition on UBE2D3 levels in wild-type ESCs undifferentiated and differentiated for 3 days to PrE, compared with ESCs differentiated to ectoderm (Ect), mesoderm (Mes), and endoderm (End) as described in Materials and Methods. Top panel: western blot and bottom panel: quantification of UBE2D3 levels after 5 h treatment of the cells with 50 μg/ml cycloheximide (mean ± SEM, *n* = 3).

To test whether the *Ube2d3*-S138A mutation affects PrE differentiation, we induced wild-type and mutant ESCs to differentiate into XEN cells, which are an in vitro model for PrE, by incubating them with all-trans retinoic acid and activin (Niakan et al. 2013; Tomaz et al. 2017). After 8 days of induction, wild-type and revertant ESCs gave rise to large numbers of cells with the characteristic XEN cell morphology, whereas the mutant cells failed to differentiate efficiently and gave significantly reduced numbers of XEN cells (fig. 5*A*). The reduced efficiency of differentiation was confirmed by a time-lapse analysis from days 4 to 8 of the differentiation, which showed greatly reduced rates of XEN cell colony formation during differentiation of the S138A mutant cells compared with the wild-type cells (supplementary video 1, Supplementary Material online).

The FGFR and PDGFRα RTKs are known to have key roles in promoting differentiation and survival of PrE cells (Molotkov et al. 2017; Bessonnard et al. 2019). Fluorescence-activated cell sorting (FACS) analysis of PDGFRα and immunofluorescent staining for PDGFRα and FGFR1 showed strong reductions in the levels of both RTKs in the mutant cells after 8 days of differentiation, confirming the impact of the *Ube2d3*-S138A mutation on PrE differentiation (fig. 5*B* and *C*). The effect of the S138A mutation on the ability of ESCs to differentiate into the three germ layers, ectoderm, mesoderm, and endoderm, was also tested (see Materials and Methods). Differentiation into the germ layers showed that wild-type and mutant ESCs gave rise to all three cell types with similar efficiency (supplementary fig. S4, Supplementary Material online).

The role of UBE2D3 phosphorylation in PrE differentiation was further tested by comparing the levels of UBE2D3-S138Ph and the stability of the UBE2D3 protein in PrE and in differentiated germ layer cells. Western blotting with anti-UBE2D3-S138Ph antibody showed an increase in the level of phosphorylated UBE2D3 from day 0 to day 3 and day 8 of PrE differentiation that was accompanied by a decrease in total UBE2D3 levels (fig. 5*D*). No increase in UBE2D3-S138Ph was observed in differentiating ectoderm and mesoderm, although an increase was observed in differentiating endoderm (fig. 5*E*). Western blotting with an anti-Aurora B-T232ph antibody, which detects the active phosphorylated form of Aurora B, showed that the level of the activated kinase was increased in differentiating PrE compared with ESCs and was not affected by the *Ube2d3*-S138A mutation (supplementary fig. S5*B*, Supplementary Material online). Comparison of the effects of cycloheximide treatment of 3-day differentiated PrE cells with treatment of differentiating ectoderm, mesoderm, and endoderm showed that UBE2D3 is less stable in PrE cells compared with germ layer cells (fig. 5*F*). Overall, these results show that there is an increase in the level of activated Aurora B and UBE2D3-S138Ph in extraembryonic PrE, which is associated with downregulation of UBE2D3 levels in these cells. Treatment of ESCs and 3-day differentiated PrE cells with the proteasomal inhibitor MG132 showed a strong increase in UBE2D3 levels, indicating that turnover of UBE2D3 protein is mediated by proteasomal degradation in both cell types (supplementary fig. S5*A*, Supplementary Material online).

### Interaction between CBL and Its RTK Substrates Is Enhanced by the *Ube2d3*-S138A Mutation

Because of the number of E3 ligases that use UBE2D3 as an ubiquitin donor and the complications that this would have caused for global proteomic approaches to analyzing the effects of the S138A mutation, we used a candidate approach to identify E3 ligases and ubiquitination targets that mediate the effects of UBE2D3-S138 phosphorylation on early embryonic development. The CBL E3 ubiquitin ligase plays an important role in regulating RTK turnover by monoubiquitinating phosphorylated RTKs, including PDGFRα, epidermal growth factor receptor, and FGFR, and promoting trafficking of the receptors to the lysosome for degradation (reviewed by Haglund and Dikic [2012]). FGFR1 and PDGFRα are involved in differentiation and survival of PrE (Molotkov et al. 2017; Bessonnard et al. 2019) and we have shown that the levels of both receptors are reduced in PrE cells differentiated from *Ube2d3*-S138A ESCs (fig. 5*B* and *C*). CBL uses UBE2D3 as an ubiquitin donor, leading us to speculate that phosphorylation of UBE2D3-S138 might be affecting the level of RTKs in PrE cells by downregulating the activity of CBL.

**Fig. 6.**
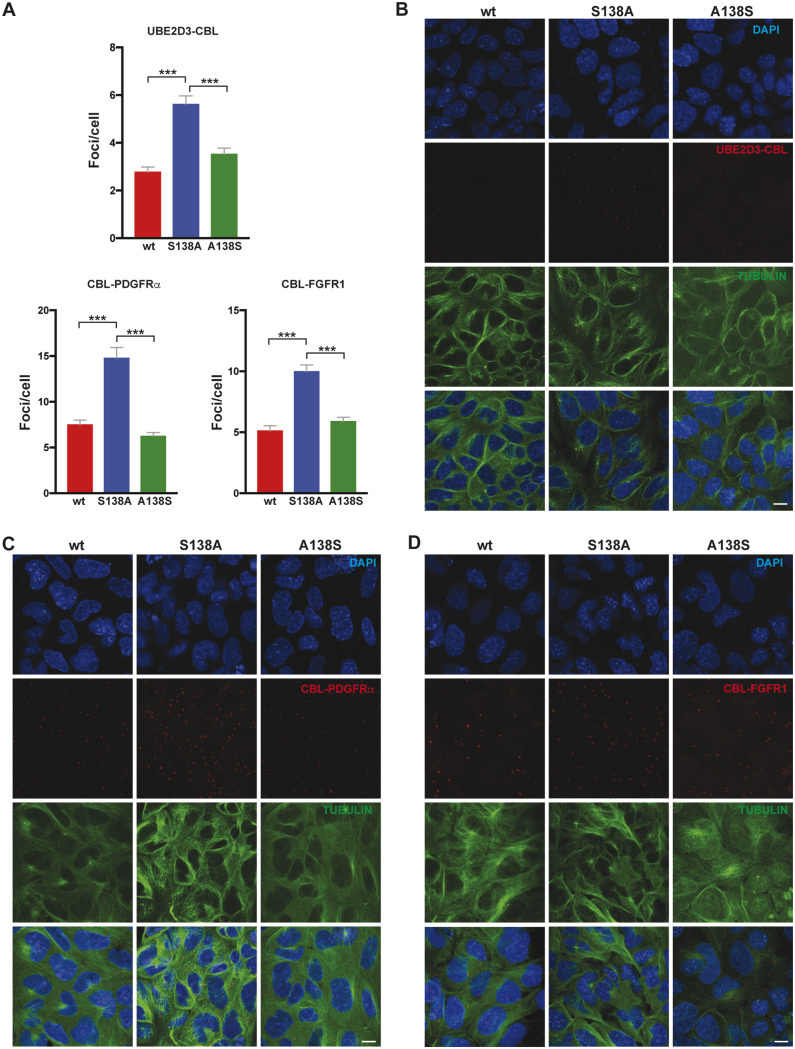
PLA of UBE2D3-CBL, CBL-PDGFRα, and CBL/FGFR1 contacts at day 3 of XEN differentiation. (*A*) Quantification of the number of PLA foci/cell, all graphs represent mean ± SEM of three biological replicates. (*B*–*D*) Representative images of PLA detected interaction between UBE2D3-CBL (*B*), CBL-PDGFRα (*C*), and CBL-FGFR1 (*D*). *P* values calculated by *t*-test; ***P < 0.001.

The proximity ligation assay (PLA) was used to measure the interaction between CBL and UBE2D3 during differentiation of ESCs into XEN (PrE) cells (fig. 6*A* and *B*). PLA detects and amplifies interactions between oligonucleotide tags on antibodies that are specific for each protein, generating interaction foci that can be visualized by microscopy. When PLA was used to compare the level of interaction in wild-type, S138A, and revertant A138S ESCs after 3 days of differentiation, a substantial increase in the number of interaction foci was observed in the S138A mutant cells compared with wild-type cells, whereas the A138S revertant cells showed a similar level of interaction to the wild-type cells (fig. 6*B*). These results provide evidence that phosphorylation of UBE2D3-S138 downregulates CBL activity during PrE differentiation and that this regulation is blocked by the S138A mutation.

PLA was also used to analyze the interaction between CBL and the RTK PDGFRα after 3 days of XEN cell differentiation. The results showed an increase in the number of interaction foci in S138A mutant cells compared with wild-types cells (fig. 6*A* and *C*). A similar increase was also observed in the interactions between CBL and FGFR1 (fig. 6*A* and *D*). The increased interactions provide an explanation for the reduction in the levels of PDGFRα and FGFR1 observed by immunofluorescence staining in differentiating *Ube2d3*-S138A PrE cells compared with wild-type and A138S revertant cells (fig. 5*B* and *C*). Treatment of 3-day differentiated wild-type PrE cells with the lysosomal inhibitor chloroquine resulted in increased levels of FGFR1 and PDGFRα (supplementary fig. S5*C*, Supplementary Material online) confirming that the levels of the receptors were being regulated by lysosomal turnover in response to monoubiquitination (Haugsten et al. 2008; Mohapatra et al. 2013). Quantitative reverse transcription PCR (RT-qPCR) analysis of the mRNA expression levels for CBL, FGFR1, and PDGFRα showed that there was no difference in mRNA levels for the three proteins in wild-type, mutant, and revertant cells (supplementary fig. S6, Supplementary Material online), confirming that that the changes in receptor levels were occurring at the posttranslational level.

UBE2D3 acts an ubiquitin donor for a number of E3s in addition to CBL. Cyclin D1 is a known target of UBE2D3-mediated ubiquitination, which leads to its downregulation (Hattori et al. 2007; Mittal et al. 2011). Analysis of Cyclin D1 levels in the S138A mutant ESCs showed that the level was significantly reduced in the mutant cells (supplementary fig. S5*D*, Supplementary Material online). As a further test of the broad effects of the S138A mutation, the level of monoubiquitination of histone H2AK119 (H2AK119Ub) was measured at gene promoters in ESCs. H2AK119 ubiquitination is mediated by the E3 ligase activity of the polycomb protein RING1B using UBE2D3 as an ubiquitin donor and has been shown to be important for maintaining poising of developmentally regulated promoters in ESCs (Stock et al. 2007). Chromatin immunoprecipitation analysis was carried out on chromatin isolated from wild-type, mutant, and revertant ESCs using primers for a range of silent and active promoters. The results show a clear increase in the level of H2AK119Ub across the majority of promoters analyzed (supplementary fig. S7*A* and *B*, Supplementary Material online) and a reduction in binding of S5-phosphorylated RNA polymerase II (RNA Pol II-S5Ph) (supplementary fig. S7*C*, Supplementary Material online). Interestingly, we observed increased H2AK119Ub and reduced RNA Pol II-S5Ph at active as well as silent promoters. This is consistent with a previous report that RING1B binds to and ubiquitinates active as well as silent promoter regions in ESCs (Brookes et al. 2012). Cyclin D1 and total H2AK119Ub levels were similar in the wild-type mutant and revertant cells in 3-day differentiated PrE cells (supplementary fig. S5*E* and *F*, Supplementary Material online), suggesting that the main role of ubiquitination of these substrates at this stage of development is likely to be in lineage priming in cells of the inner cell mass. Overall, our results show that the increased UBE2D3 levels caused enhanced ubiquitination by a number of different E3s. This highlights the broad effect that phosphorylation of UBE2D3-S138 is likely to have on ubiquitination in amniote species.

## Discussion

Amniotes first appeared during the Carboniferous period between 314 and 340 Ma (Carroll 1964; Clack 2012) and ultimately became the dominant vertebrate life forms on dry land. The results of this study identify an alanine to serine substitution that occurred in the common ancestor to modern amniotes, generating a novel mechanism for regulating the promiscuous ubiquitin-conjugating enzyme UBE2D3. The conservation of the alanine residue at position 138 in UBE2D3 from single-celled eukaryotes through to modern invertebrate metazoa and anamniote vertebrates, together with the absolute conservation of the serine residue in amniotes, provides strong evidence that the substitution was a highly unusual event that happened only once in eukaryotic evolution. Acquisition of the serine created a phosphorylation site for the cell cycle kinase Aurora B, and we have shown that phosphorylation of this site drastically reduces the stability of the UBE2D3 protein by altering the position of the C-terminal α-helix and exposing the hydrophobic core of the protein. This causes a reduction in the solubility of the protein, targeting it for degradation by the proteasome.

Experiments in mice and ESCs showed that phosphorylation of UBE2D3-S138 is required for mouse embryonic development. Intriguingly, the strongest effect of the mutation that we observed in ESCs and embryos was on the differentiation of extraembryonic endoderm lineages (PrE and VE), which are known to have important roles in the formation of the amniote extraembryonic membranes and in lineage specification in the early embryo. Parietal endoderm derived from the PrE forms the inner lining of the yolk sac, which is one of the most ancient of the extraembryonic structures in amniotes (Stewart 1997; Ferner and Mess 2011). The yolk sac mediates nutritional exchange in the early embryonic stages of oviparous and viviparous amniotes (Ferner and Mess 2011; Sheng and Foley 2012). VE, which is also derived from PrE, plays essential roles in the positioning of the primitive streak during gastrulation in chick and mouse embryos (Stern and Downs 2012).

The experiments using PLA provide clear evidence that the S138A mutation affects the functioning of the E3 ligase CBL, with increased interaction between UBE2D3 and CBL and between CBL and two of its target RTKs, FGFR1 and PDGFRα, observed during differentiation of S138A mutant ESCs into XEN cells. Ubiquitination by CBL is essential for mouse development, with mice that are null for the two major isoforms c-CBL and CBL-b showing an embryonic lethal phenotype before E10.5 (Naramura et al. 2002). Our results provide evidence that modulation of the E3 ligase activity of CBL by downregulation of UBE2D3 is also essential for mouse embryonic development and formation of VE, and we show that the increased CBL activity resulting from higher levels of UBE2D3 in the *Ube2d3*-S138A mutant cells is associated with reduced levels of FGFR1 and PDGFRα in differentiating XEN (PrE) cells.

**Fig. 7.**
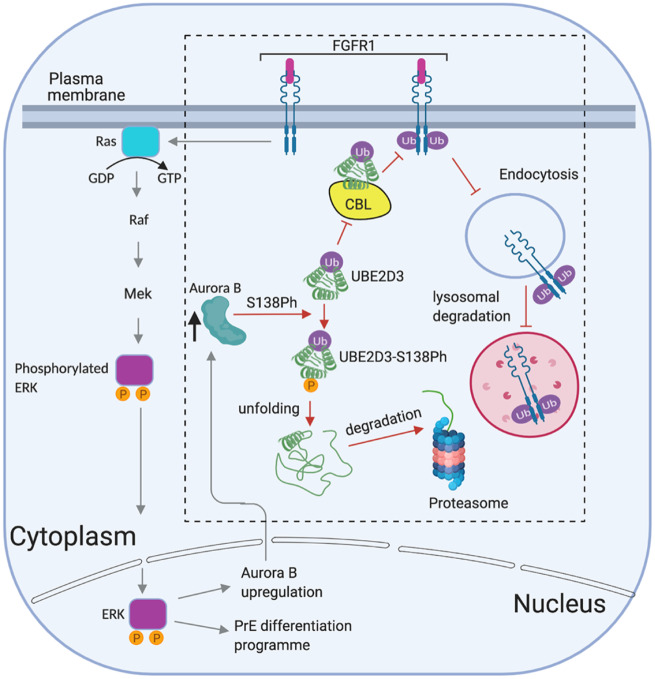
Model to explain the role of UBE2D3 phosphorylation in the differentiation of PrE. The model shows the proposed effect of phosphorylation of UBE2D3-S138 by Aurora B on signaling through the FGFR1 RTK. The dashed box delineates pathways that have been shown in this study to be directly affected by UBE2D3 phosphorylation. Activation of FGFR1 by binding of FGF to the receptor triggers a feedback mechanism through monoubiquitination of FGFR1 by the CBL E3 ligase (Levkowitz et al. 1999), using UBE2D3 as an ubiquitin donor. Monoubiquitination promotes lysosomal localization and degradation of FGFR1 following internalization of the activated receptor (Haugsten et al. 2008). Phosphorylation of UBE2D3-S138 by Aurora B downregulates UBE2D3 levels, reducing CBL activity. This in turn reduces lysosomal degradation of FGFR1, thereby enhancing FGFR1 signaling and promoting PrE differentiation. Signaling through FGFR1 activates the RAS/MEK/ERK pathway leading to increased levels of phosphorylated ERK in the nucleus. Activation of the ERK pathway has been shown to increase Aurora B expression in melanoma cells (Bonet et al. 2012). This suggests a potential positive feedback mechanism, whereby ligand-mediated activation of FGFR1 would increase Aurora B levels through activation of the ERK pathway. This would, in turn, suppress CBL activity by reducing the level of UBE2D3, further increasing the level of FGFR1. Reduced CBL activity would also increase the level of PDGFRα, which has been shown to promote survival and proliferation of PrE (Bessonnard et al. 2019).

A model illustrating the role that we propose for UBE2D3-S138 phosphorylation in PrE differentiation is shown in figure 7. A notable feature of this model is that it suggests that phosphorylation of the UBE2D3-S138 residue has the potential to generate a positive feedback mechanism that would amplify signaling through FGFR1 and PDGFRα in differentiating PrE cells. Activation of the RAS/MEK/ERK pathway by FGF signaling has been shown to be required for differentiation of PrE in mouse embryos (Hamilton and Brickman 2014). The ERK pathway has been shown to have the capacity to upregulate Aurora B expression in cancer cells and primary T cells (Song et al. 2007; Bonet et al. 2012). In differentiating PrE cells, this would have the effect of reducing UBE2D3 protein levels through phosphorylation of S138, downregulating CBL activity. This would, in turn, upregulate FGFR1, resulting in further upregulation of Aurora B and downregulation of the inhibitory role of CBL/UBE2D3 on FGFR1 in a positive feedback loop. This mechanism would also amplify the levels of PDGFRα, which has been shown to be important for survival and proliferation of PrE (Bessonnard et al. 2019).

A number of different E3s are known to use UBE2D3 as an ubiquitin donor (Brzovic and Klevit 2006; Marblestone et al. 2013), which suggests that ubiquitination of additional protein targets is likely to be affected by UBE2D3 phosphorylation. Direct evidence for this comes from our observation of increased ubiquitination of histone H2AK119 at repressed and active genes and downregulation of Cyclin D1 levels in *Ube2d3*-S138A ESCs. Both of these proteins are known targets for ubiquitination using UBE2D3 as an ubiquitin donor. These findings suggest that UBE2D3 phosphorylation could affect other biological processes in embryonic and adult tissues in addition to the effects on RTK activity and PrE differentiation mediated by CBL. Further studies will be required to determine the nature and extent of these effects. Overall, our observations reveal an unexpected role for UBE2D3 in the evolution of extraembryonic membranes in the stem amniote lineage and indicate that variation in ubiquitin signaling can act as a significant mechanism for evolutionary change.

## Materials and Methods

### Comparative Analysis of UBE2D3 Amino Acid Sequences

The comparison of UBE2D3 protein sequences from vertebrates was based on protein sequences downloaded from the UCSC 100-way MULTIZ alignment for hg19 exons against 100 vertebrate species. Protein sequences were checked manually for accuracy using gene sequences from the UCSC browser, NCBI, or ENSEMBL (see below). *Ube2d3* genes were identified by the presence of an additional minor alternative exon (exon-7a) that lies upstream from the predominantly used exon-7b and is specific to UBE2D3 (see supplementary Methods, Supplementary Material online). Manually annotated fish UBE2D3 sequences, in particular, differed significantly from the sequences produced by automated annotation. This is probably due to the high homology between UBE2D3 family members. The UBE2D3 sequence for the Carolina anole lizard was obtained by transcriptomic mining, and the two partial candidate UBE2D3 sequences from Axolotl were identified by blasting the Axolotl genome. UBE2D3-like protein sequences from nonvertebrate eukaryotes were identified by obtained by blasting the UniProt database. Full details of the approaches used to identify *Ube2d3* genes and verify the protein sequences can be found in supplementary Methods, Supplementary Material online. Sources and sequence coordinates or accession numbers for UBE2D3 orthologs are shown in supplementary table 2, Supplementary Material online.

### Mice

All work involving mice was carried out under the regulations of the British Home Office and was approved by the Imperial College Animal Welfare and Ethical Review Body. A detailed protocol on the generation of UBE2D3 mutant embryos is described in supplementary Methods, Supplementary Material online.

### Cells

E14 ESCs (female, 129/Ola) were obtained from the LMS Stem Cells and Transgenics facility. ESCs were maintained on gelatin coated plates, at 37 °C, 5% CO_2_, in KnockOut Dulbecco’s Modified Eagle’s Medium supplemented with 15% fetal bovine serum, 1× NEAA, 2 mM l-glutamine, 100 units/ml penicillin, 100 μg/ml streptomycin, 100 μM βME (all reagents from ThermoFisher), and 1,000 units/ml LIF (Merck). For differentiation experiments and cell treatments, see supplementary Methods, Supplementary Material online.

### UBE2D3 Gene Editing Using CRISPR/Cas9

To produce E14 *Ube2d3*-S138A mutant ESC, 4 × 10^6^ cells were transfected with 3 μg of pX330 (Cong et al. 2013; Addgene plasmid #42230) plasmid carrying the appropriate gRNA, 4 μg of the donor ssDNA and 3 μg of a puromycin resistance plasmid (pCAG-puro^R^) using the Mouse ES Cell Nucleofector Kit (Lonza). One day after transfection, cells were subjected to puromycin selection (1.5 μg/ml) for 24 h. A week after transfection, individual clones were picked and genotyped by nested PCR. Mutant genotypes were confirmed by sequencing. For details of the gRNAs and donor sequences used in this study, see supplementary Methods, Supplementary Material online.

### Immunofluorescence

Detailed protocols for whole-mount embryo immunostaining and cell immunofluorescence are described in supplementary Methods, Supplementary Material online. The primary and secondary antibodies used in this study are listed in supplementary table 6, Supplementary Material online. Images were acquired in a Leica SP8 confocal microscope with LAS X software (Leica) or in an Olympus IX70 microscope. The images were analyzed with Fiji software (Schindelin et al. 2012).

### Proximity Ligation Assay

PLAs were performed with Duolink in situ PLA (Sigma, mouse and rabbit probes) following the manufacturer’s instructions. A step by step protocol can be found in supplementary Methods, Supplementary Material online.

### Transient Expression of UBE2D3 Mutant Proteins in ESCs

Wild-type *Ube2d3* and *Ube2d3*-S138A, -S138E, and -S138D mutants were cloned into the expression vector pCAG-IRES-GFP (Addgene, plasmid #11159). E14 cells (5 × 10^6^) were transfected with 10 μg of the appropriate plasmid with the Mouse ES Cell Nucleofector Kit (Lonza) and allowed to grow for 48 h after transfection. Cells were then collected and sorted for GFP expression. UBE2D3 protein levels were analyzed by western blot.

### Protein Expression in Bacteria and Extraction

The cDNAs of *Ube2d3* mutants (wild-type, S138A, S138E, and S138D) were cloned into the pET-N-His (Origene) vector containing a 6× His N-terminal tag. *Escherichia coli* BL21(DE3) cells (Agilent) were transformed with the pET- *Ube2d3* plasmids, and protein expression was induced with 0.4 mM IPTG (Merck) for 3 h at 37 °C. Bacteria were pelleted and incubated in lysis buffer (50 mM Tris–HCl pH 8.0, 150 mM NaCl, 10 mM imidazole, 7 mM β-mercaptoethanol, 300 μg/ml lysozyme) for 30 min at 4 °C. The lysate was then sonicated and spun (15,000 × g, 10 min, 4 °C). The supernatant was collected as soluble fraction, and the pellet was subjected to the same lysis. The pellet obtained after the second lysis-sonication was resuspended in lysis buffer, sonicated one more time, and collected as the insoluble fraction.

### Molecular Modeling

The UBE2D3 structure was retrieved from the Protein Data Bank (PDB: 3UGB) (Page et al. 2012), along with the conjugated ubiquitin. Two systems were set up: one with the original wild-type structural and one with a phosphorylation on the Ser138 side chain. Ubiquitin was maintained on both simulations but excluded from the analysis of results. The phosphorylated Ser138 (Sep138) model was obtained by submitting the original structure to the CHARMM-GUI server (Jo et al. 2008, 2014). Parameters to describe the thioester bond between UBE2D3 and ubiquitin were generated by submitting a dipeptide composed by the two residues that participate in this interaction (Gly and Cys) to the CGenFF server (Vanommeslaeghe et al. 2010; Yu et al. 2012) and adapted into the CHARMM36 force field. Details of the methodology for the Molecular Dynamic Simulations can be found in supplementary information, Supplementary Material online.

### Statistical Analysis

All statistical analyses were performed with Prism software. The statistical tests used in each experiment and significances are indicated in the corresponding figure legends.

### Genotyping, Chromatin Immunoprecipitation-qPCR, RNA Extraction, Flow Cytometry, In Vitro Phosphorylation, and Ubiquitination Assays and Western Blot

For details of these procedures, see supplementary Methods, Supplementary Material online.

## Supplementary Material

Supplementary data are available at *Molecular Biology and Evolution* online.

## Supplementary Material

msaa060_Supplementary_Data
