# Supplementary material for: Evolution of an Amniote-Specific Mechanism for Modulating Ubiquitin Signaling via Phosphoregulation of the E2 Enzyme UBE2D3
Source: Mol Biol Evol. 2020 Mar 12;37(7):1986–2001. doi: 10.1093/molbev/msaa060 (PMC7306689; doi:10.1093/molbev/msaa060)
Supplement: msaa060_Supplementary_Data [file msaa060_supplementary_data.zip › msaa060-Suppl_Data/Supplementary Information.pdf]

Roman-Trufero et al.

Supplementary Information

Figure S1

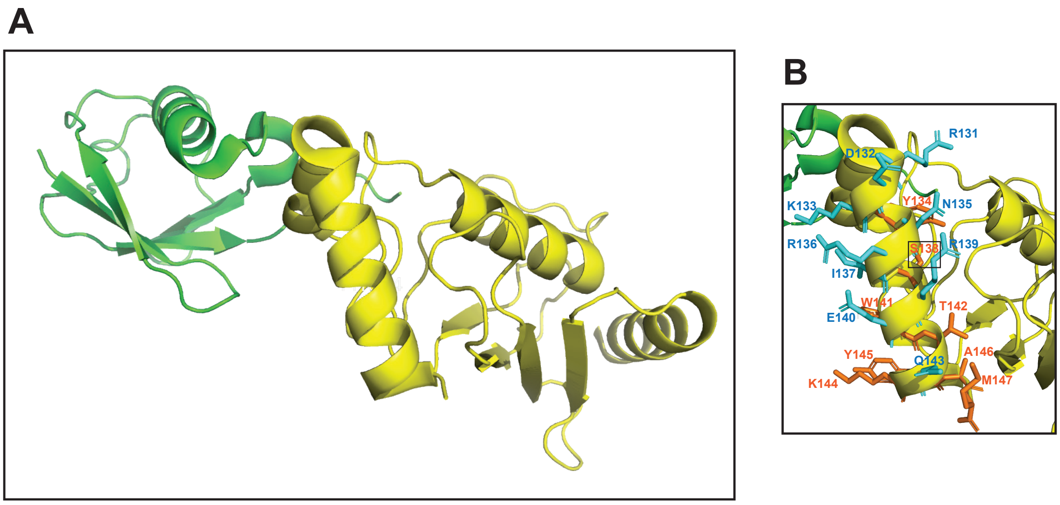

Figure S1. Structure of the human UBE2D3-ubiquitin conjugate. (A) UBE2D3 is shown in yellow and the conjugated ubiquitin molecule is coloured green. (B) Close up of the C-terminal α4-helix showing the side-chains of residues 131-147. Side-chains of variable residues are shown in cyan and side-chains of residues that are largely invariant are coloured orange. S138 (indicated by a box) is classified as largely invariant as it changes only once during evolution, at the anamniote/amniote transition. Position 138 is occupied by serine in amniotes and alanine in anamniotes and non-vertebrate eukaryotes. The images were prepared by visualising PDB 3UGB using PyMOL (v1.8.6.0.).

Figure S2

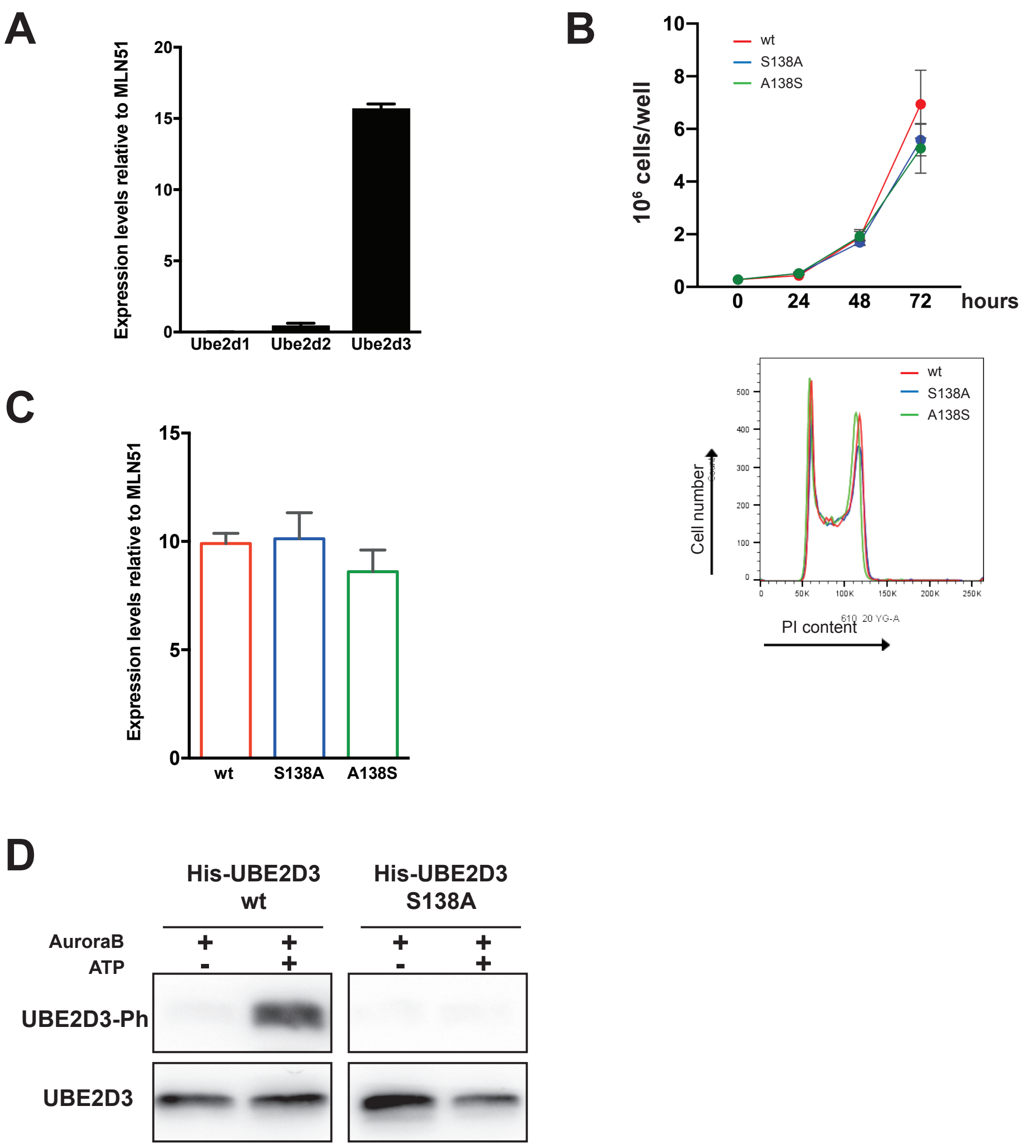

Figure S2. Characterisation of the *Ube2d3*-S138A mutant and A138S revertant ESCs. (A) qRT-PCR analysis of RNA levels for UBE2D family members in ESCs. Transcripts were quantified using expression of the *Mln51* gene as a standard (Szutorisz et al. 2005). (B) Top panel, proliferation assay: cells were plated at 10000 cells/cm^2 density and allow to grow for 3 days. Bottom panel: Analysis of cell cycle profiles; wild-type, mutant and revertant cells were stained with PI and analysed by FACS. (C) qRT-PCR analysis of *Ube2d3* mRNA in wild-type, S138A mutant and A138S revertant ESCs. Transcripts were quantified relative to *Mln51*. (D) Specificity of the anti-UBE2D3-S138Ph (UBE2D3-Ph) antibody. His-tagged

UBE2D3 and UBE2D3-S138A were expressed in bacteria, purified and incubated with Aurora B kinase and ATP. Western blotting was carried with anti-UBE2D3-Ph (upper panels) and anti-UBE2D3 (lower panels). All graphs represent mean ± SEM, n=3.

Figure S3

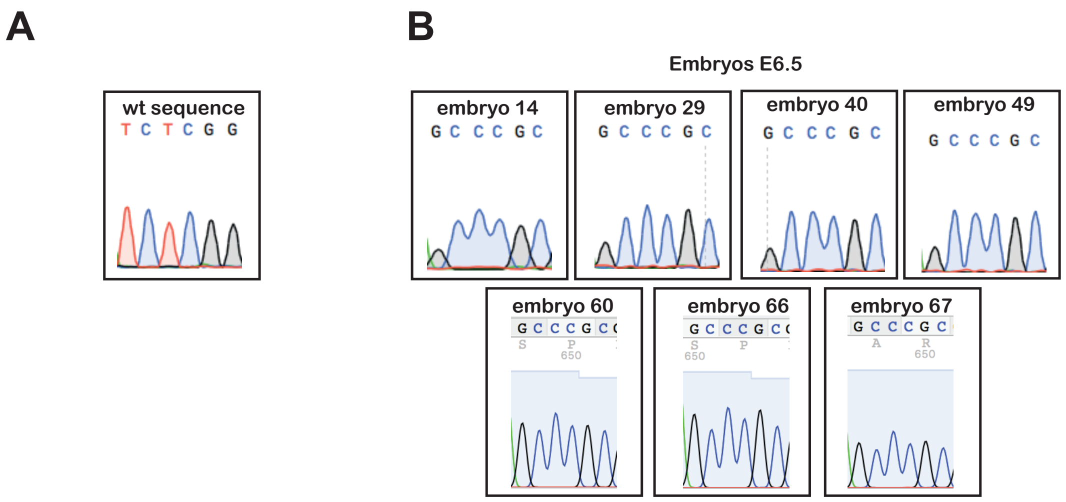

Figure S3. Sequences obtained from E6.5 embryos genotyped as homozygous for the *Ube2d3*-S138A mutation by PCR. (A) Wild-type (wt) sequence. (B) Sequences from homozygous S138A mutant E6.5 embryos. The sequencing confirmed that all seven E6.5 embryos were homozygous.

Figure S4

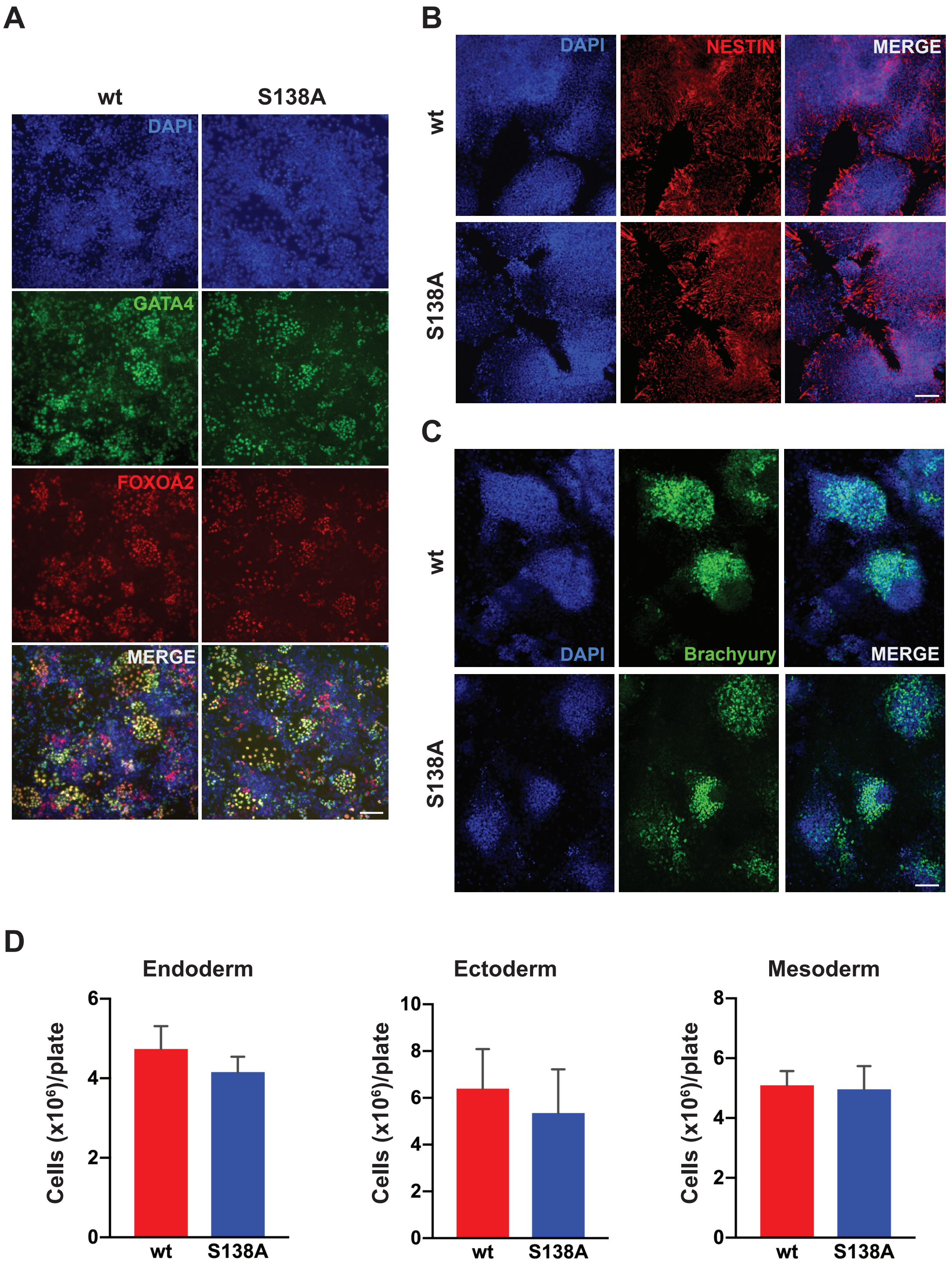

Figure S4. Differentiation to somatic lineages of wt and *Ube2d3*-S138A ESCs. (A) Differentiation of ESCs into endoderm. Wt and S138A cells were stained with anti-Gata4 and anti-FoxA2 after 3 days of differentiation. (B) Differentiation of ESC into ectoderm. Wt and S138A cells were stained with anti-Nestin antibody after 4 days of differentiation. (C) Differentiation of wt and S138A ESCs into mesoderm. Cells were stained with anti-Brachyury antibody after 4 days of differentiation. All scale bars = 100 μm. (D) Quantification of the final number of cells per plate in the differentiation experiments (mean ± SEM, n=3).

Figure S5

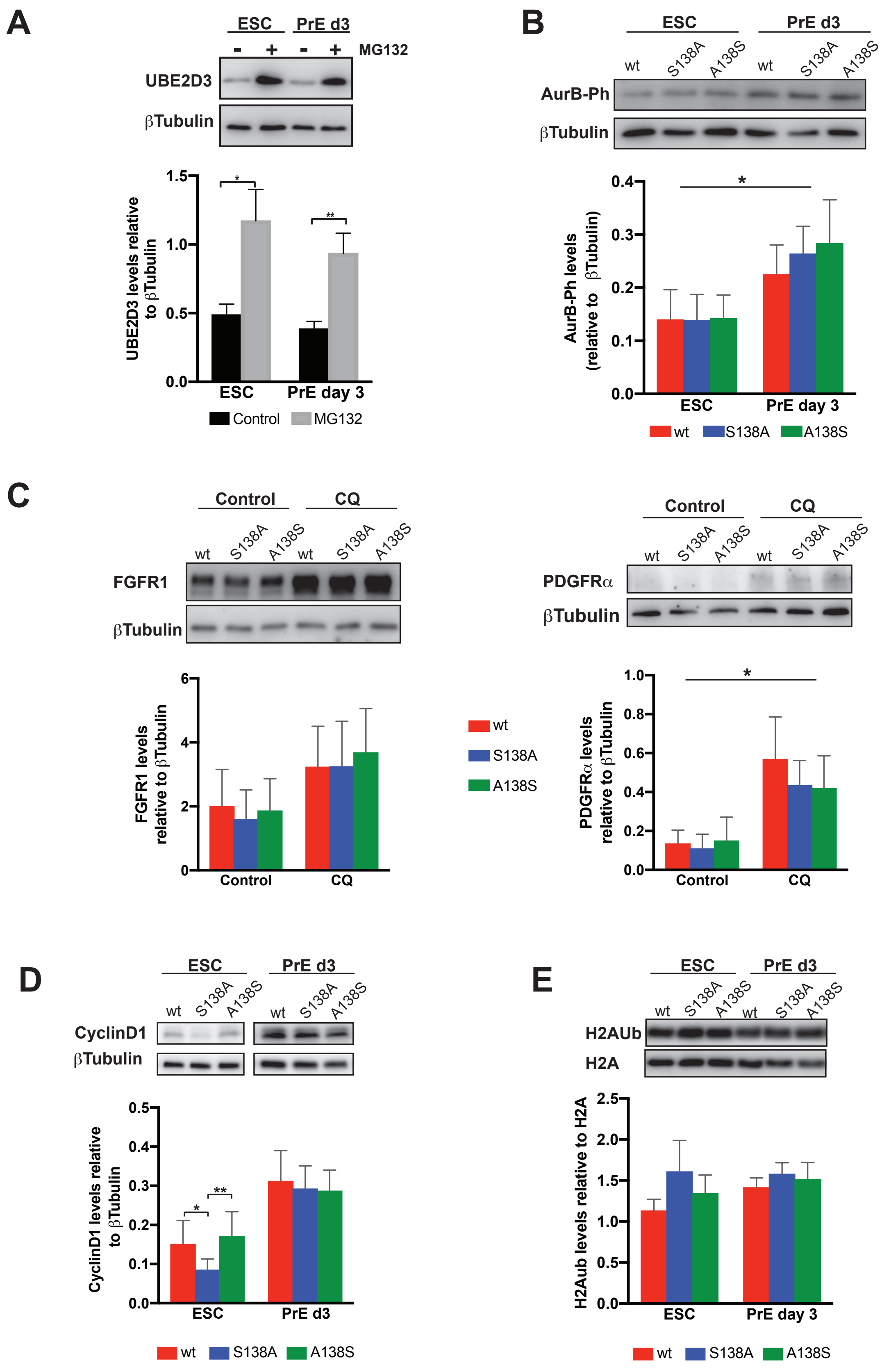
A
ESC
PrE d3
- + - + MG132
UBE2D3
βTubulin
UBE2D3 levels relative to βTubulin
1.5
1.0
0.5
0.0
*
**
ESC
PrE day 3
Control
MG132
B
ESC
PrE d3
wt S138A A138S wt S138A A138S
AurB-Ph
βTubulin
AurB-Ph levels (relative to βTubulin)
0.4
0.3
0.2
0.1
0.0
*
ESC
PrE day 3
wt
S138A
A138S
C
Control
CQ
wt S138A A138S wt S138A A138S
FGFR1
βTubulin
FGFR1 levels relative to βTubulin
6
4
2
0
Control
CQ
wt
S138A
A138S
Control
CQ
wt S138A A138S wt S138A A138S
PDGFRα
βTubulin
PDGFRα levels relative to βTubulin
1.0
0.8
0.6
0.4
0.2
0.0
*
Control
CQ
D
ESC
PrE d3
wt S138A A138S wt S138A A138S
CyclinD1
βTubulin
CyclinD1 levels relative to βTubulin
0.5
0.4
0.3
0.2
0.1
0.0
*
**
ESC
PrE d3
wt
S138A
A138S
E
ESC
PrE d3
wt S138A A138S wt S138A A138S
H2AUb
H2A
H2Aub levels relative to H2A
2.5
2.0
1.5
1.0
0.5
0.0
ESC
PrE day 3
wt
S138A
A138S

Figure S5. Analysis of protein levels in ESCs and differentiating cells. (A) Western blot analysis of wt ESCs and cells from day 3 of primitive endoderm differentiation (PrE d3) after treatment with the proteasome inhibitor MG132 for 4 hours. Bottom panel, quantification of UBE2D3 levels relative to β-Tubulin (mean ± SEM, n=3, p-values calculated by t-test, *p<0.05, ** p<0.01). (B) Western blot analysis of Aurora B-T232Ph (AurB-Ph) levels in ESCs and cells from day 3 of differentiation into primitive endoderm (PrE). Bottom panel, quantification of AuroraB-Ph levels relative to β-Tubulin (mean ± SEM, n=4, p-value calculated by Two-way ANOVA, *p<0.05). (C) Western blot analysis of cells on day 3 of differentiation to PrE treated with 10 μM chloroquine (CQ) for 16 hours. Left panel, western blot and quantification of FGFR1 levels (mean ± SEM, n=3). Right panel, western blot and quantification of PDGFRα levels (mean ± SEM, n=3, p-value calculated by Two-way ANOVA, *p<0.05). (D) Western blot analysis of Cyclin D1 in ESCs and cells from day 3 of differentiation into primitive endoderm (PrE). Bottom panel, quantification of Cyclin D1 levels relative to β-Tubulin (mean ± SEM, n=4). (E) Western blot analysis of H2AUb levels on ESC and cells from day 3 of differentiation to primitive endoderm (PrE). Bottom panel, quantification of H2Aub levels relative to H2A (mean ± SEM, n=4).

Figure S6

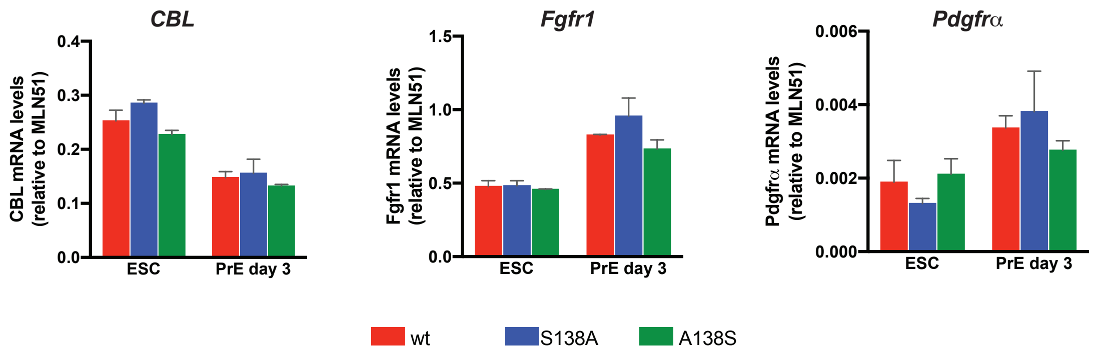

Figure S6: Gene expression analysis in wild-type ESCs, undifferentiated and on day 3 of differentiation to primitive endoderm (PrE). qRT-PCR analysis of *CBL* (left), *Fgfr1* (middle) and *Pdgfrα* (right) transcripts. Quantification was carried out using expression of the *Mln51* gene as a standard (mean ± SEM, n=3).

Figure S7

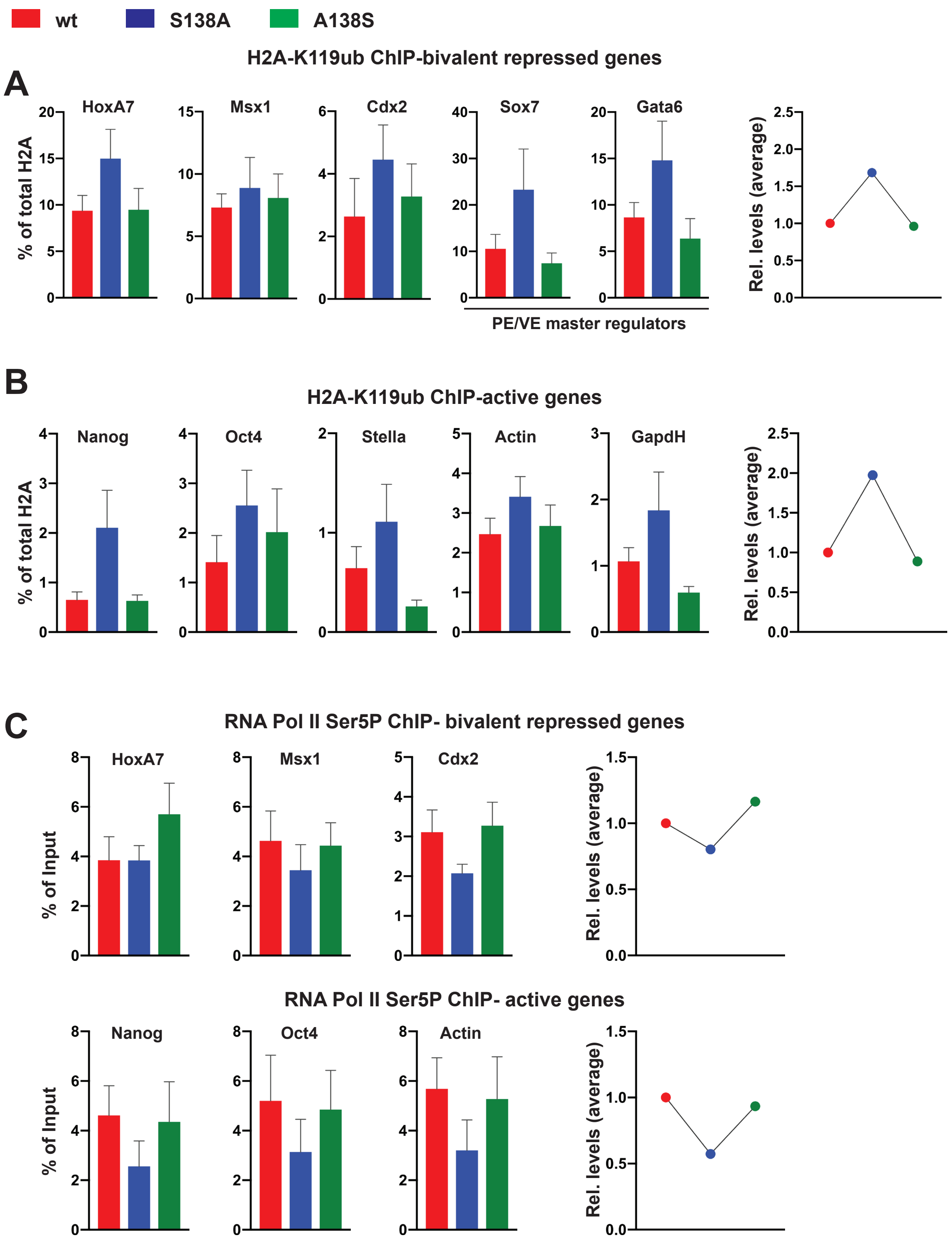

Figure S7. Changes in H2AK119Ub and Ser5Pol II levels in promoters of *Ube2d3*-S138A mutant ESCs. (A) ChIP-qPCR analysis of H2AK119Ub levels on bivalent promoters in wild-type (wt), *Ube2d3*-S138A mutant and *Ube2d3*-A138S revertant ESCs (n=6). (B) ChIP-qPCR analysis of H2AK119Ub levels in active genes promoters in wt, S138A and A138S ESCs (n=6). (C) ChIP-qPCR analysis of Ser5Pol II levels in bivalent promoters (top) and active genes (bottom) in wt, S138A and A138S ESCs. Graphs for individual promoters represent mean ± SEM, n=5 for Msx1 and Actin, n=6 for the other genes. Graphs on the right in each section represent the mean levels at the analysed promoters.

Supplementary Table 5. Oligonucleotides used in this study.

<table>
<tr><th></th><th>Forward</th><th>Reverse</th></tr>
<tr><td colspan="3">Genotyping primers</td></tr>
<tr><td>1st PCR</td><td>TGCTGGCTGTTTGAAGAGGG</td><td>CCAGGCAGCTAACTCATTGGT</td></tr>
<tr><td>2nd PCR wt specific</td><td>GTTTGTTTACAGGTACAACAGAATATCT</td><td rowspan="2">ATTCCAGCTATAATGCAGGTTATTC</td></tr>
<tr><td>2nd PCR S138A specific</td><td>ATCGTCTAAGGTTTGTTTACAGGTACAACAGAATAGCC</td></tr>
<tr><td colspan="3">ChIP-qPCR primers</td></tr>
<tr><td>Msx1</td><td>ACAGAAAGAAATAGCACAGACCATAAGA</td><td>TTCTACCAAGTTCCAGAGGGACTTT</td></tr>
<tr><td>HoxA7</td><td>GAGAGGTGGGCAAAGAGTGG</td><td>CCGACAACCTCATACCTATTCCTG</td></tr>
<tr><td>Cdx2</td><td>GGACTCCGCGAGCCAA</td><td>CTCAGCCCACGGTGCTC</td></tr>
<tr><td>Sox7</td><td>TTTAGGGAAGTCAGTGCGCC</td><td>AGATCACACCCATGGCTTGG</td></tr>
<tr><td>Gata6</td><td>GTTTCCCTCCCTCTTCTGCC</td><td>CGCTGTAACACATCCCCAGT</td></tr>
<tr><td>Nanog</td><td>CACAGTTTGCCTAGTTCTGAGG</td><td>GCAAGAATAGTTCTCGGGATGAA</td></tr>
<tr><td>Oct4</td><td>GGCTCTCCAGAGGATGGCTGAG</td><td>TCGGATGCCCCATCGCA</td></tr>
<tr><td>Stella</td><td>AGAGCGGGGAATCCTACAGT</td><td>ACTGTAGGATTCCCCGCTCT</td></tr>
<tr><td>GapdH</td><td>CCACTTGTGGCAAGAGGCTA</td><td>GTGGAGAGTTGGGACGTGAG</td></tr>
<tr><td>β-Actin</td><td>GCAGGCCTAGTAACCGAGACA</td><td>AGTTTTGGCGATGGGTGCT</td></tr>
<tr><td colspan="3">Expression analysis primers</td></tr>
<tr><td>Ube2d1</td><td>GCAACCATCATGGGGCCCCC</td><td>CAAGACAAATACTCCCGTTG</td></tr>
<tr><td>Ube2d2</td><td>TTCCACCATGGCTCTGAAGA</td><td>CTCCGCCCTGATAGGGACTA</td></tr>
<tr><td>Ube2d3</td><td>ACAGACTATGGCGCTGAAAC</td><td>TCCCATAATTGTGGCTTGCC</td></tr>
<tr><td>FGFR1</td><td>AGGGCAACTACACCTGCATC</td><td>CAAGTTGTCTGGCCCGATCT</td></tr>
<tr><td>PDGFRα</td><td>CCTGGCGCAAGGAAAAATTGT</td><td>CCAGAGCAGAATGCCATAGGA</td></tr>
<tr><td>CBL</td><td>CGTGGCAGGAATCAGAAGGT</td><td>CTTGTGGGGCCATGGAGAAT</td></tr>
<tr><td>MLN51</td><td>ATGACGATGAGGATCGGAAAAAC</td><td>GTCCCCTTGGGTCGGACTTC</td></tr>
</table>

Supplementary Table 6. Antibodies used in this study.

Antibody	Source	Application	Dilution
UBE2D3	Abcam (Cat# ab176568)	WB	1:3000
UBE2D3-Ph	Frangini *et al.* 2013	WB	1:750
H3	Abcam (Cat# ab1791)	WB	1:10000
βTubulin	Cell Signaling (Cat# 2146)	WB	1:2000
HA	Cell Signaling (Cat# 3724)	WB	1:1000
Aurora B-Ph	Cell Signalling (Cat# 2914)	WB	1:1000
CyclinD1	Cell Signaling (Cat# 2922)	WB	1:2000
GFP	Cell Signaling (Cat# 2555)	WB	1:2000
H2A	Abcam (Cat# ab182255)	WB, ChIP	1:2000, 2.5 μg
H2AUb	Cell Signaling (Cat# 8240)	WB, ChIP	1:2000, 2.5 μg
Ser5Ph-RNA PolII	Biolegend (Cat# 904001)	ChIP	2.5 μg
IgG	Diagenode (Cat# C15410206)	ChIP	2.5 μg
Oct4	Santa Cruz (Cat# sc-5279x)	IF	1:200
Gata6	R&D Systems (Cat# AF1700)	IF	1:100
FoxoA2	Abcam (Cat# ab108422)	IF	1:400
Gata4	Santa Cruz (Cat# sc-1237)	IF	1:300
Nestin	Abcam (Cat# ab11306)	IF	1:200
Brachyury	Santa Cruz (Cat# sc-17743)	IF	1:500
PDGFRa-PE	eBioscience (Cat# 12_1401)	Flow Cyt.	1:100
PDGFR	Cell Signaling (Cat# 3174)	IF, PLA	1:500
FGFR1	Cell Signaling (Cat# 9740)	IF, PLA	1:200
CBL	Novus (Cat# NBP 2-37574)	PLA	1:200
Tubulin-FITC	Sigma (Cat# F2168)	PLA	1:50
Alexa Fluor 488 Donkey anti goat	Invitrogen (Cat# A11055)	IF	1:500
Alexa Fluor 555 Donkey anti mouse	Invitrogen (Cat# A31570)	IF	1:500
Alexa Fluor 488 Donkey anti rabbit	Invitrogen (Cat# A32790)	IF	1:500
Alexa Fluor 594 Donkey anti rabbit	Invitrogen (Cat# R37119)	IF	1:500
Goat anti mouse-HRP	Invitrogen (Cat# A16084)	WB	1:10000
Goat anti rabbit-HRP	Invitrogen (Cat# A16096)	WB	1:10000

SUPPLEMENTARY METHODS:

Comparative analysis of UBE2D3 amino acid sequences

Annotation of the *UBE2D3* gene can present problems for automated gene annotation systems because of the high level of homology between different UBE2D family members. To address this problem, we made use of the fact that *UBE2D3* in vertebrates has an alternative C-terminal exon (designated here as exon 7a, with amino acid sequence in amniotes: YNRLAREWTEKYAML) that is located between exon 6 and the predominantly expressed downstream exon 7b (sequence: YNRISREWTQKYAM). Exon-7a is not expressed in mouse ESCs (data not shown). The fact that exon-7a is not present in other UBE2D family members and can be readily distinguished from the downstream C-terminal exon-7b by the presence of the additional lysine residue at the C-terminus allowed us to use exon-7a as a diagnostic feature to verify the identity of the *UBE2D3* genes that we used for the vertebrate species comparison. All of the vertebrate *UBE2D3* genes used to generate the sequences shown in fig. 1 and Supplementary Tables 1 and 2 were individually checked for the presence of exon-7a except for the sequence from *Anolis carolinensis*, which was obtained by transcriptome mining (see below). Species where it was not possible to identify Exon-7a (Axolotl and Medaka) are discussed below.

Quality assessment of the *ube2d3* sequences from fish

Due to the variable quality of the annotations of available fish genome sequences and the high level of sequence variation that was observed in the C-terminal α-helix in a subset of teleost species, all of the fish sequences shown in Figure 1 and Supplementary Table 1 were checked manually by downloading the gene sequences, translating them and checking the sequences of the *ube2d3* exons. Where variant residues were found at intron/exon boundaries, the splice junctions were also checked manually to ensure that the variation was not caused by incorrect alignment of the splice sites. Files used for the sequence verification are available on request.

A number of the *UBE2D3* sequences from amniotes and anamniotes also lacked the distally located amino terminal exon (MALKRINK in amniotes). Blast searching using available first exon sequences was used to search for the exon. In some species, it was not possible to locate the first exon (see Table S2). This is likely to be due to gaps in the available sequences.

Annotation of the *ube2d3* sequence in the Carolina anole lizard (*Anolis carolinensis*).

We first used the Ensembl and UCSC gene annotation to check the status of position 138 of UBE2D3 in *Anolis carolinensis* and found that both databases annotated this position as alanine in the Anocar2.0 genome assembly. However, the ENSEMBL and UCSC databases rely on the first generation of gene annotation for the anole genome, which is primarily based on sequence homology with related species. This could lead to problems with the annotation due to the very high sequence conservation between *ube2d3* and *ube2d2*. The fact that all of the other non-avian-reptile species and all avian species examined have serine at position 138 also suggested that the presence of the alanine might be due to a misannotation. We therefore carried out a further analysis using a new genome

reannotation of *Anolis carolinensis* based on 14 adult and embryonic deep transcriptome (Eckalbar, et al. 2013). Our new analysis based on transcriptomic data in Eckalbar et al. shows that *ube2d3* with serine in position 138 is one of the genes that is poorly represented in the Anocar2.0 assembly because the contig with *ube2d3* cannot be mapped to Anocar2.0 with a high mapping rate. The second-generation annotation for *A. carolinensis* ASU_Acar version 2.1 identified the *ube2d3* transcript as a8840. The sequence and ID of this transcript are currently available at NCBI (https://www.ncbi.nlm.nih.gov/nuccore/GAFD01029227.1). The transcript encodes a protein that is 100% identical to human UBE2D3 with serine at position 138. We conclude that the location predicted by the Ensembl and UCSC gene annotation as *ube2d3* is in fact *ube2d2* with alanine at position 138. We also conclude that *ube2d3* with serine at position 138 is expressed in *A. carolinensis* but was not annotated in Anocar2.0 because of incomplete genome assembly.

<u>Identification of *ube2d3* in Axolotl (*Ambystoma mexicanum*)</u>
The exceptionally large size of Urodele genomes (up to 10 times larger than humans) and the high levels of repetitive sequences in these genomes have made them difficult to sequence. An annotated genome sequence for Axolotl has recently become available (Nowoshilow, et al. 2018) and this allowed us to carry out a blast search for Axolotl *ube2d3* using the exon-6 and exon-7 sequences from the Tibetan frog (*Nanorana parkeri*). The blast search identified two UBE2D genes (for gene co-ordinates, see Supplementary Table 2). Using the co-ordinates, we were able to obtain the full sequence of exon-6 and exon-7 and the intron that separates them for both genes from the UCSC genome browser (https://genome.axolotl-omics.org). The intron was searched for the presence of the alternatively spliced exon-7a, but the alternative exon was not present in either gene sequence. The sequence of Exon-6 shows clearly that the genes are UBE2D2/3 and not UBE2D1/4. Examination of the intron sequence also shows that they are not duplicated as there is a ~3-fold difference in the length of the non-repetitive sequence in the introns of the two genes. We conclude from this that we have identified the *ube2d2* and *ube2d3* genes of Axolotl but the absence of the alternative Exon-7a does not allow us to say definitively which of the genes is *ube2d3*. The sequence of Exon-7 differs between the two genes by one residue (A or E) at position 132. Therefore, the sequence of Axolotl *ube2d3* Exon-7 is shown as ambiguous for these residues in fig. 1 and Supplementary Tables 1. The absence of Exon-7a could be due to the exon having been lost during Axolotl evolution or it could be caused by a gap in the sequence.

<u>Medaka *ube2d3*-b:</u> It was not possible to locate Exon-7a in one of the two UBE2D3-like sequences that were identified in Medaka. However, the sequence of the Medaka gene was identical to the *ube2d3*-b gene of Zebra mbuna and Pundamilla nyererei and was therefore identified as a bona-fide *ube2d3* gene and was designated as Medaka *ube2d3*-b (fig. 1 and Supplementary Table 1)

<u>Giant Panda (*Ailuropoda melanoleuca*):</u> The annotated *Ube2d3* sequence for the Giant Panda contains a cluster of 4 variant residues at positions 112, 113, 115, 116. Since 3 of these residues are invariant in all other eukaryotes examined, we concluded that the apparent variation in the Giant Panda sequence was the

result of a sequencing error. Therefore, the Giant Panda was not included in the sequence comparison.

Sequences from non-vertebrate eukaryotes. Protein sequences of UBE2D3 homologues from representatives of non-vertebrate eukaryotic lineages were mined from the Uniprot database by blasting the entire database with conserved sequences from amino-terminal and C-terminal regions of *Drosophila melanogaster* Effete/UBCD1 that did not include position-138. The sequences used for the blast were: MALKRINKEL; REWTRKYA; REWTRKYAM. Sequences were also obtained by examination of protein sequences of individual E2 conjugating enzymes from model organisms in the Uniprot database.

UBE2D3 gene editing using CRISPR/Cas9

The guide RNA (gRNA) used to generate the Serine to Alanine mutation was 5'-
CACCGCAGGTACAACAGAATATCTC-3' and the gRNA used to revert the mutation was 5'-CACCGACAACAGAATAGCCCGCGAA-3'.
The donor ssDNAs used in this study are: 5'-
TTAATTTTATTTTTTAAATAGCTTATTTGTTTGTTTACAGGTACAACAGAATAGCCC
GCGAATGGACTCAGAAGTATGCCATGTGATGCTACCTTACAGTCAGAATAACC-3'
carrying the S138A mutation; 5'-
TTAATTTTATTTTTTAAATAGCTTATTTGTTTGTTTACAGGTACAACAGAATATCTC
GCGAATGGACTCAGAAGTATGCCATGTGATGCTACCTTACAGTCAGAATAACC-3' to re-establish the wild type sequence and 5'-
TTAATTTTATTTTTTAAATAGCTTATTTGTTTGTTTACAGGTACAACAGAATAAGCC
GCGAATGGACTCAGAAGTATGCCATGTGATGCTACCTTACAGTCAGAATAACC-3' to introduce a Serine to Serine synonymous mutation.

To generate mouse embryos with the *Ube2d3*-S138A mutation, gRNA for the mutation was transcribed *in vitro* and injected into C56Bl6/CBA F1 fertilized eggs together with Cas9 mRNA and the donor ssDNA. To obtain E6.5 and E12.5 embryos, the injected embryos were transplanted into foster mothers and harvested at the appropriate time point.

Genotyping of point mutations in mouse ESCs and embryos

To genotype the point mutations, we used a nested allele-specific PCR (ASPCR), approach (Gaudet, et al. 2009). The mutated region was first amplified in a normal 15-cycle PCR. An aliquot of 1 μl from the first PCR was then subjected to a 25 cycle, 3-primer ASPCR with a common reverse primer and two genotype-specific forward primers. Both products can be discriminated due to a 10 base tail at the 5' end of the mutant specific primer. The final PCR products were run on a 3% MetaPhor™ agarose gel. The primers used for the genotyping are described in Supplementary Table 5.

ESC culture

ESC differentiation into XEN cells: Differentiation was performed using the protocol detailed by (Niakan, et al., 2013). For time-lapse imaging of differentiation, cells were transferred on day 4 of differentiation to a Zeiss Axiovert 200 microscope (Zeiss) where images were taken at 10 minute intervals until day 8. Images were analysed using Volocity software (Perkin Elmer).

ESC differentiation to endoderm: 10000 cells/cm^2 cells were plated on gelatin-coated plates and incubated in high glucose DMEM with 15% FBS, 100 units/ml penicillin, 100 μg/ml streptomycin, 0.1 mM non-essential amino acids, 1mM MTG, 1x GlutaMAX and supplemented with 25 ng/ml of FGF2 and 10 μM of retinoic acid for 3 days (Kim, et al. 2010).
ESC differentiation to mesoderm: cells were plated in gelatin + fibronectin-coated plates at a density of 15000 cells/cm^2 and incubated for 4 days in DMEM/F12 N2B27, 1x GlutaMAX, 100 units/ml penicillin, 100 μg/ml streptomycin, 0.1% β-ME and 30 ng/ml of Activin A.
ESC differentiation to ectoderm: cells were plated at a density of 15000 cells/cm^2 on gelatin-coated plates and incubated for 4 days in DMEM/F12-Neurobasal medium (50/50), 1x GlutaMAX, 100 units/ml penicillin, 100 μg/ml streptomycin, 100 μM βME, B27 minis Vitamin A and N2.
Aurora B inhibition: ESCs were incubated for 3 hours with 200 nM AZD1152 (Merck) or DMSO in medium.
Protein synthesis inhibition: ESC were incubated for 1h and 5h with 50 μg/ml of cycloheximide (Cell Signalling Technology) or DMSO in medium.
Proteasome inhibition: ESCs were incubated for 4 hours with 5 μM of MG132 (Cell Signalling Technology).
Lysosome inhibition: ESCs and 3 day differentiated XEN cells were incubated for 16 hours with 10 μM of chloroquine (Sigma).

Flow cytometry

All experiments were performed using an LSRII flow cytometer (Becton-Dickinson) and analysed with FlowJo Software. Cells were harvested, washed twice with PBS and incubated at a densitiy of 10^6 cell/ml with anti-PDGFRα (1:100) in 2%FBS-PBS for 20 min at 4°C. After incubation, cells were washed twice, resuspended in 2%FBS-PBS at $5x10^5$ cells/ml and analysed.
Cell cycle analysis: ESCs were trypsinised, washed twice with PBS, fixed with 70% ethanol for 30 min on ice and washed twice with 2% FBS-PBS. Finally cells were resuspended in PBS containing 1μg/ml RNAse A (ThermoFisher), 50 μg/ml propidium iodide and 0.05% NP40, incubated 20 min at room temperature in the dark followed by analysis.

Proximity Ligation Assay

Cells were fixed with 4% PFA at 37°C during 15 min, washed 3x5 min with PBS, permeabilized with 0.4% Tx-100 in PBS for 5 min at room temperature and blocked 1 hour at 37°C with blocking solution. Incubation with the primary antibodies was performed at 4°C overnight (the antibodies used and their dilutions are indicated in Supplementary Table 6). The following day, cells were washed 2x5 min with buffer A, incubated with the minus and plus probes for 1h at 37°C in antibody diluent solution, washed 2x5 min with buffer A, incubated with Duolink ligation mix 30 min at 37°C, washed 2x5 min with buffer A, incubated with Duolink amplification mix 1h and 40 min at 37°C, washed 2x10 min with buffer B, 1x5 min with buffer A and counterstained with anti-Tubulin FITC for 1h at room temperature. After counterstaining, cells were washed 2x2 min with buffer A, 1x1 min with 0.01x buffer B and incubated 5 min with 1μg/ml DAPI in PBS. All incubations were performed in a humidity chamber. Images were acquired in a Leica SP8 confocal microscope with LAS X software (Leica),

PLA foci were quantified with Imaris software (Oxford Instruments). Technical control experiments were carried out in which each primary antibody was omitted. No signal was observed in these controls (data not shown).

Whole-mount immunostaining

Embryos were harvested at the specified time points and fixed in 4% paraformaldehyde + 0.01% Triton -100 + 0.1% Tween for 20 min at 4°C. Fixed embryos were washed 3 times for 5 min in 0.1% Triton-PBS (PBT) at room temperature. Embryos were then blocked and permeabilised in blocking solution (2% donkey serum-0.4% Triton X-100/PBS) for 2 hours at room temperature. Incubation with the primary antibodies was carried out at 4°C, overnight for blastocysts and for 2 days for E6.5 embryos, with the primary antibodies diluted in blocking solution. Embryos were then washed 3 x 15 min in PBT, incubated with the appropriate secondary antibodies diluted in blocking solution for 2 hours at room temperature, washed again 3 times 15 min in PBT and left overnight in PBT+1μg/ml DAPI at 4°C until imaging the next day.

Cell immunostaining

Cells were fixed with 4% PFA at 37°C during 15 minutes, after this time they were washed 3 x 5 min with PBS. Cells were then permeabilised with 0.4% TX-100 in PBS for 5 min at room temperature and blocked with 10% donkey serum-0.1% Tx-100-PBS. Incubation with the primary antibodies was carried out overnight at 4°C. The cells were then washed 3 x 5 min with PBS and incubated 1h at room temperature with the appropriate secondary antibodies. Cells were washed 3 x 5 min and incubated with 1μg/ml DAPI in PBS for 5 min.

ChIP-qPCR analysis

ESCs were harvested, counted and diluted in medium to a final density of 10^6 cells/ml prior to being crosslinked for 10 min at room temperature with 1% formaldehyde. Crosslinking was quenched by adding glycine to 125 mM final concentration and incubating for 10 min at room temperature. After crosslinking, cells were washed 3 times with cold PBS. The resulting pellet was resuspended in 1.5 ml of swelling buffer (25 mM Hepes pH 7.5, 1.5 mM $MgCl_2$, 10 mM KCl, 0.1% NP40, Complete™ protease inhibitors cocktail), incubated in ice for 10 min and then homogenized in a dounce homogenizer (tight pestle). The nuclei were spun for 5 min at 3000g, 4°C and then resuspended in sonication buffer (50 mM Hepes pH7.5, 140 mM NaCl, 1 mM EDTA, 1% Triton-X100, 0.1% sodium deoxycholate, 0.1% SDS, Complete™ protease inhibitors cocktail) at a final density of $20x10^6$ cells/ml. Sonication was performed in a BioruptorPlus sonicator (Diagenode) for 30 to 45 min with 30 seconds on/off intervals to produce fragments of approximately 0.5-1 kb. Sonicated material was centrifuged for 10 min at 10000g, 4°C, to remove the insoluble fraction and supernatants were collected and snap frozen. Chromatin concentration and shearing of DNA were analysed by reverse crosslinking of an aliquot and running on an agarose gel.

Aliquots of 10 μg were used per immunoprecipitation (IP) and 1 μg used as input. For each IP, 2.5 μg of antibody (anti-H2A, anti H2Aub, anti Ser5RNAPolII or IgG) were incubated together with 15 μl protein A/G Dynabeads (ThermoFisher) in 1 ml of sonication buffer for 5 to 7 hours with gentle rotation at 4°C. Beads were washed once with sonication buffer and then the chromatin

was added, IPs were performed O/N at 4°C with gentle rotation in a final volume of 1 ml. Antibody-bound chromatin was washed sequentially with 1 ml of the following buffers: sonication buffer, high salt buffer (50 mM Hepes pH 7.5, 500 mM NaCl, 1 mM EDTA, 1% Triton-X100, 0.1% sodium deoxycholate, 0.1% SDS), LiCl buffer (20 mM TriHCl pH8, 1mM EDTA, 250 mM LiCl, 0.5% NP40, 0.5% sodium deoxycholate) and TE buffer (10 mM TrisHCl pH8, 1 mM EDTA). After the washes, the chromatin was eluted from the beads and crosslinking was reversed by incubation in 200 µl of elution buffer (50 mM TrisHCl pH8, 50 mM NaCl, 1 mM EDTA, 1% SDS, 20 µg/ml DNAse-free RNAse A) for 6 hours at 68°C. Eluted DNA was treated O/N with 200 µg/ml proteinase K, at 42°C. Reverse crosslinking of the input was performed in parallel to that of the beads. DNA from input and IPs was purified using the QIAQuick PCR purification kit (Qiagen) following the manufacturer's instructions and eluted in 30 µl of water.
Real-time qPCRs were performed with Sensimix SYBR NORox SYBR GREEN (Bioline, UK) in a C1000 Thermal Cycler (BioRad). Each PCR was carried out in duplicate in a 20 µl final volume reaction with 400 nM primer concentration. Reactions contained 1 µl of the IPed DNA and 0.1 µl of the input DNA.
The oligonucelotides used for ChIP are listed in Supplementary Table 5.

In vitro phosphorylation assay
Recombinant His-UBE2D3 (1 µg) was incubated with 200 ng of GST-AuroraB (MRC PPU, College of Life Sciences, University of Dundee, Scotland, mrcppureagents.dundee.ac.uk) for 30 min at 30°C in phosphorylation buffer (50 mM TrisHCl pH 7.5, 10 mM $MgCl_2$, 500 µM ATP, 1 mM DTT, 5 mM NaF). Reactions were stopped by addition of SDS loading buffer, boiled for 5 min or used for subsequent ubiquitination assays. Phosphorylation was analysed by western blot.
For the kinase and spin assay, in vitro phosphorylation reactions were performed as indicated above and the spun for 15 min at 15000g at 4°C, washed 2x with phosphorylation buffer and subsequently analysed by western blot.

Protein analysis by western blotting
Cells were lysed in ice cold RIPA buffer (50 mM TrisHCl pH 8, 150 mM NaCl, 1% NP40, 0.5% sodium deoxycholate, 0.1% SDS) with Complete™ protease inhibitor cocktail (Roche) for 10 min, sonicated (MSE Soniprep150, 3 times 5 sec on/1 min off, 14 µm amplitude) and cleared by centrifugation for 10 min at 14000g. Concentration of the extracts was measured with the BCA Protein Assay Kit (ThermoFisher) according to the manufacturer's instructions. Extracts were diluted in 6x SDS loading buffer, boiled for 5 min and subjected to SDS PAGE. Gels were transferred to nitrocellulose membranes using a Bio-Rad wet tank blotting system. Membranes were blocked with 5% bovine serum albumin (BSA) in Tris-buffered saline with 0.05%NP40 (TBS-NP40) for 1h at room temperature and incubated with the primary antibody diluted in 0.5% BSA TBS-NP40 O/N at 4°C. On the following day, membranes were washed 3 times for 20 min with TBS-NP40, incubated with the appropriate secondary antibody for 1h at room temperature, washed again 3 times 15 min and developed using Millipore Crescendo ECL (Merck). For western blot quantification, membranes were developed and analysed using the ImageQuant LAS400mini and the ImageQuantTL Software (GE Healthcare). The antibodies used are listed in Supplementary Table 6.

In the case of western blots probed with the anti-UBE2D3-S138Ph antibody, membranes were denatured prior to blocking by incubating the membranes for 30 min at 4°C with 6M guanidine hydrochloride, 20 mM Tris HCl pH 7.4, 1mM PMSF and 5 mM βME solution.

RNA extraction and qPCR

Total RNA was extracted from $1x10^6$ cells using Trizol (ThermoFisher) following the manufacturer's instructions. Reverse transcription was performed on 1 μg of RNA for each sample with the SuperScript II reverse transcriptase (ThermoFisher) according to the manufacturer's protocol. To quantify the levels of UBE2D family members mRNA, Real-time qPCRs were performed on the obtained cDNA using Sensimix SYBR NORox SYBR GREEN (Bioline). Each PCR was carried out in duplicate using 1 μl of a 1:10 dilution of cDNA and 400 nM primer concentration. The primers used in this analysis are listed in Supplementary Table 5.

Molecular modelling

Atomistic Molecular Dynamics Simulations**.**

Simulations were performed using the GROMACS simulation suite (2018.3 version) (Abraham et al., 2015), the CHARMM36 force field (Huang and MacKerell 2013) and the TIP3P water model (Jorgensen et al., 1983). Simulations were performed for 200 ns each, with two independent replicates for each system (800 ns, overall). The chosen temperature was 340 K in order to enhance sampling and denaturation of the structures. It was maintained by making use of the velocity rescale thermostat (Bussi, et al. 2007) with a time constant of 1ps. For pressure coupling, the Parrinello-Rahman barostat (Parinello et al., 1981) was employed to keep pressure at 1 atm with a time constant of 1 ps. Particle Mesh Ewald method (Darden et al., 1993) handled the long-range electrostatics. The LINCS algorithm (Hess et al., 2008) was applied to constrain bonds, allowing an integration step of 2 fs. Long-range electrostatic and van der Waals cut-offs were set at 1.4 nm. Charges were neutralized by the addition of counterions and an additional 0.15 M concentration of NaCl ions were included in the simulations. VMD (Humphrey, et al. 1996) was employed for molecular visualization, and analyses were performed using GROMACS tools.

SUPPLEMENTARY DISCUSSION:

Effects of the whole genome duplication in teleosts

It is known that a whole-genome duplication took place in the common ancestor to all teleosts ~320-350 mya (Christoffels, et al. 2004; Vandepoele, et al. 2004). Genome duplications can have one of several different effects on the evolution of individual genes. One of the duplicated copies can be lost, both genes can remain the same, both genes can acquire sequence changes that divide the functions of the ancestral gene between them (subfunctionalization), or one gene can diverge and acquire a new function (neofunctionalization) (reviewed in (Glasauer and Neuhauss 2014). In this study, duplicated *ube2d3* genes were initially identified in Medaka, Southern platyfish and Stickleback. The pattern of variation that was observed in the *ube2d3* genes that were initially identified in Cichlid fish did not

follow the known phylogeny of Cichlids, suggesting that there might be additional unidentified duplicated *ube2d3* genes in these species. Blasting of Cichlid genomes with variant C-terminal sequences show that this was indeed the case. The blasting identified additional duplicated *ube2d3* genes in two Cichlid species (Zebra mbuna and Pundamilia nyererei). The duplicated genes are designated *ube2d3*-a and –b (fig. 1, Supplementary Tables 1 and 2). These 5 species were the only Acanthomorph species where duplicated *ube2d3* genes could be confirmed, but it is likely that the presence of these genes is more widespread. More comprehensive sequencing will be required to confirm this. Comparison of the sequences reveals a significant divergence between of the duplicated genes in the C-terminal encoding region (Supplementary Table 1) with the variable sequences located predominantly on the outward-facing side of the α4-helix where they would be available for protein-protein contacts (supplementary fig. 1B). This suggests that sub-functionalization or neo-functionalization involving the formation of novel protein-protein contacts are potential options for the protein products of duplicated *ube2d3* genes.

References

Abraham, M.J., Murtola, T., Schulz, R., Páll, S., Smith, J.C., Hess, B and Lindhal, E. (2015). GROMACS: High performance molecular simulations through multi-level parallelism from laptops to supercomputers. SoftwareX *1*, 19-25.
Bussi G, Donadio D, Parrinello M. 2007. Canonical sampling through velocity rescaling. J Chem Phys 126:014101.
Christoffels A, Koh EG, Chia JM, Brenner S, Aparicio S, Venkatesh B. 2004. Fugu genome analysis provides evidence for a whole-genome duplication early during the evolution of ray-finned fishes. Mol Biol Evol 21:1146-1151.
Darden, T., York, D. and Pedersen, L. (1993). Particle mesh Ewald: An N log(N) method for Ewald sums in large systems. J Chem Phys *98*, 10089.
Eckalbar WL, Hutchins ED, Markov GJ, Allen AN, Corneveaux JJ, Lindblad-Toh K, Di Palma F, Alfoldi J, Huentelman MJ, Kusumi K. 2013. Genome reannotation of the lizard Anolis carolinensis based on 14 adult and embryonic deep transcriptomes. BMC Genomics 14:49.
Gaudet M, Fara AG, Beritognolo I, Sabatti M. 2009. Allele-specific PCR in SNP genotyping. Methods Mol Biol 578:415-424.
Glasauer SM, Neuhauss SC. 2014. Whole-genome duplication in teleost fishes and its evolutionary consequences. Mol Genet Genomics 289:1045-1060.
Hess, B. (2008). P-LINCS: A Parallel Linear Constraint Solver for Molecular Simulation. J Chem Theory Comput *4*, 106-122
Huang J, MacKerell AD, Jr. 2013. CHARMM36 all-atom additive protein force field: validation based on comparison to NMR data. J Comput Chem 34:2135-2145.
Humphrey W, Dalke A, Schulten K. 1996. VMD: visual molecular dynamics. J Mol Graph 14:33-38, 27-38.
Jorgensen, W.L., Chandrasekar, J., Madura, J.D., Impey, R.W. and Klein, M.L. (1983). Comparison of simple potential functions for simulating liquid water. J Chem Phys *79*, 926-935.
Kim PT, Hoffman BG, Plesner A, Helgason CD, Verchere CB, Chung SW, Warnock GL, Mui AL, Ong CJ. 2010. Differentiation of mouse embryonic stem cells into endoderm without embryoid body formation. PloS one 5:e14146.

Nowoshilow S, Schloissnig S, Fei JF, Dahl A, Pang AWC, Pippel M, Winkler S, Hastie AR, Young G, Roscito JG, et al. 2018. The axolotl genome and the evolution of key tissue formation regulators. Nature 554:50-55.
Parrinello, M. and Rahman, A. (1981). Polymorphic transitions in single crystals: a new molecular dynamics method. J Appl Phys *52*, 7182-7190.
Szutorisz, H., Canzonetta, C., Georgiou, A., Chow, C-M, Tora, L., Dillon, N. Formation of an active tissue–specific chromatin domain initiated by epigenetic marking at the ES cell stage. Mol. Cell. Biol. 25: 1804-1820.
Vandepoele K, De Vos W, Taylor JS, Meyer A, Van de Peer Y. 2004. Major events in the genome evolution of vertebrates: paranome age and size differ considerably between ray-finned fishes and land vertebrates. Proc Natl Acad Sci U S A 101:1638-1643.
